# The unfolded protein response plays dual roles in rice stripe virus infection through fine-tuning the movement protein accumulation

**DOI:** 10.1371/journal.ppat.1009370

**Published:** 2021-03-04

**Authors:** Chenyang Li, Yi Xu, Shuai Fu, Yu Liu, Zongdi Li, Tianze Zhang, Jianxiang Wu, Xueping Zhou

**Affiliations:** 1 State Key Laboratory of Rice Biology, Institute of Biotechnology, Zhejiang University, Hangzhou, China; 2 State Key Laboratory for Biology of Plant Diseases and Insect Pests, Institute of Plant Protection, Chinese Academy of Agricultural Sciences, Beijing, China; 3 Department of Plant Pathology, Nanjing Agricultural University, Nanjing, China; University of California, Davis Genome Center, UNITED STATES

## Abstract

The movement of plant viruses is a complex process that requires support by the virus-encoded movement protein and multiple host factors. The unfolded protein response (UPR) plays important roles in plant virus infection, while how UPR regulates viral infection remains to be elucidated. Here, we show that rice stripe virus (RSV) elicits the UPR in *Nicotiana benthamiana*. The RSV-induced UPR activates the host autophagy pathway by which the RSV-encoded movement protein, NSvc4, is targeted for autophagic degradation. As a counteract, we revealed that NSvc4 hijacks UPR-activated type-I J-domain proteins, NbMIP1s, to protect itself from autophagic degradation. Unexpectedly, we found NbMIP1 stabilizes NSvc4 in a non-canonical HSP70-independent manner. Silencing *NbMIP1* family genes in *N*. *benthamiana*, delays RSV infection, while over-expressing *NbMIP1*.*4b* promotes viral cell-to-cell movement. Moreover, *OsDjA5*, the homologue of *NbMIP1* family in rice, behaves in a similar manner toward facilitating RSV infection. This study exemplifies an arms race between RSV and the host plant, and reveals the dual roles of the UPR in RSV infection though fine-tuning the accumulation of viral movement protein.

## Author summary

The unfolded protein response (UPR) is a cellular response aiming to eliminate abnormal proteins in and restoring ER homeostasis. The infection of many plant viruses can induce the UPR of plant cells, and previous studies show that in most cases, the UPR benefits plant viral infections. However, how the UPR regulates plant viral infections is still poorly understood. Here, we find that a plant virus, rice stripe virus (RSV), can induce host UPR, and the UPR plays dual roles in RSV infection. On the one hand, the RSV-induced UPR activates the host autophagy pathway by which the virus-encoded movement protein, NSvc4, is degraded. On the other hand, the RSV-induced UPR also upregulates the expression of a type-I J-domain protein family in plant, which is hijacked by NSvc4 to protect itself from autophagic degradation. We present an example for an arms race between RSV and the host plant, and illustrate a new model of the UPR regulating viral infection in plants by fine-tuning the accumulation of virus-encoded MP.

## Introduction

Viruses are obligate parasites which rely on their host living cell to complete their lifecycle. A fundamental problem for viruses is how to move from infected cells to their neighboring healthy cells. Plant viruses encode proteins specialized as movement proteins (MP) to help the virus’ intra- and inter-cellular spread. Plant cells are enclosed by a rigid cell wall, which makes it hard for the virus to move between cells directly. However, plant cells are connected through plasmodesmata (PD), symplastic tunnels between cells that are the gateway for cell-cell communication [1]. Many virus-encoded MPs have been reported to manipulate PD to increase their size exclusion limit (SEL), which helps the virus to travel from one cell to the next [2]. There are several types of viral MPs, among them, the model of 30 K superfamily MPs is one of the best-studied [3]. Rice stripe virus (RSV), causing one of the most devastating viral diseases of rice in East Asia [4], is a segmented negative-sense plant virus, in the *Tenuivirus* genus, family *Phenuiviridae*. The viral genome consists of four single-stranded RNA molecules, which range in size from approximately 8.9 to 2.1 kb [5,6]. The complementary genome strand of RNA4 encodes the NSvc4 protein, which was identified as a member of the 30K MP superfamily [3,5]. *Nicotiana benthamiana* is a widely used experimental host in plant virology. Previous work has shown that RSV can infect *N*. *benthamiana* by mechanical inoculation, causing symptoms including dwarfing, vein yellowing, and leaf curling [7,8]. The RSV-*N*. *benthamiana* model has since then been intensively used to study viral pathogenicity and host resistance mechanisms [8,9].

The unfolded protein response (UPR), also called endoplasmic reticulum (ER) stress response, is a cellular response that is activated by many stress conditions in the ER [10]. In plants, these stresses include environmental conditions such as drought, salinity, heat, and also biotic stresses such as pathogen invasion [11]. Unfolded proteins aggregate in the ER under these conditions will damage the normal functions of this organelle. The UPR is a comprehensive physiological process including the halting of protein translation, ER biogenesis, the degradation of misfolded proteins, and the increase in the production of molecular chaperones involved in protein folding [10]. The ultimate aim of these processes is eliminating abnormal proteins and restoring ER homeostasis [10]. Several studies have demonstrated that viral infection induces ER stress and the UPR in plant cells [12–18]. In most cases, activation of the UPR promotes the viral infection, while silencing or knocking out UPR transcription factors inhibits it [12,15,19]. To date, studies of the UPR in plant viruses mainly focus on positive-strand RNA viruses, while the effects of the UPR to plant negative-strand RNA viruses remain elusive.

Autophagy is a conserved eukaryotic cellular recycling process through degrading cytoplasmic materials. Besides nutrient recycling, autophagy is also involved in numerous physiological reactions, including cell growth, starvation, anti-aging, and defense [20,21]. As one of the main protein turnover pathways, autophagy can be activated by ER stress to eliminate unfolded proteins [22,23]. A growing body of evidence indicates that the host autophagy pathway is activated by plant viruses and plays important roles in the viral infection. Plant autophagy can degrade viral proteins by directly interacting with them [24–27]. For example, plant protein NbP3IP can direct degradation of RSV silencing suppressor protein P3 to limit virus infection through interaction with the autophagy-related protein NbATG8 [27]. As a counter-defense strategy, the barley stripe mosaic virus (BSMV) γb protein subverts tobacco autophagy by disrupting the ATG7-ATG8 interaction to promote the virus infection [28]. In some cases, viral proteins can also hijack the host autophagy machinery to target antiviral factors for degradation. For example, the RNA silencing suppressors of both poleroviruses and geminiviruses utilize the host autophagy pathway to degrade host factors involved in RNA interference to promote the viral infection [29,30]. Although some studies show that plant viruses can activate the host autophagy pathway, the molecular mechanism about how host autophagy pathway is activated and the relationship between the UPR and autophagy in viral infection are still unclear.

Besides the activation of autophagy, the UPR can also upregulate the expression levels of abundant molecular chaperones [31]. The 70-kDa heat shock protein chaperons (HSP70s) are ubiquitous molecular chaperones that act in a large variety of cellular processes [32]. A large set of co-chaperones comprising J-domain proteins and nucleotide exchange factors (NEFs) stimulate the ATPase activity of HSP70s, which is allosterically coupled to substrate binding and release [32]. J-domain proteins, also called heat shock protein 40 (HSP40) or DnaJ proteins, are co-chaperone partners of HSP70s. DnaJ proteins are named after a conserved ~70-amino-acid J domain, which has a central HPD motif (a tripeptide of histidine, proline, and aspartic acid) [32]. DnaJ proteins represent a large family of protein chaperones, which can be classified into four types (I-IV). Type I J-domain proteins contain four domains, including a J domain, a glycine/phenylalanine-rich (G/F) domain, a zinc-finger domain with 4 repeats of the motif CXXCXGXG, and a less conserved C-terminal domain (CTD). The other three types lack one or more of these domains [32]. ‘J-like proteins’, which lack the central HPD motif in the J domain, have been designated as Type IV J proteins [33]. DnaJ proteins are involved in many cellular processes and play a vital role in a variety of abiotic and biotic stress responses, primarily by stimulating the ATPase activity of chaperone proteins, HSP70s [32–36]. Multiple studies have also indicated that DnaJ proteins play important roles in plant-virus interactions. For example, the interaction between the potyvirus capsid protein and host DnaJ-like proteins is essential for the viral infection in tobacco [37]. The type-I DnaJ proteins NbMIP1s (MP-Interacting Protein 1) regulate tobacco mosaic virus (TMV) infection and R gene-mediated resistance [38].

In the present study, we show that RSV infection triggers the UPR which activates the autophagy pathway in *N*. *benthamiana*. NSvc4 is unstable in plant cell and degraded through the autophagy pathway. Moreover, the UPR also upregulates the type-I J-domain NbMIP1 proteins which are hijacked by NSvc4 to protect itself from autophagic degradation in an HSP70 chaperone-independent manner. We further show that silencing *NbMIP1* family genes delays RSV infection in *N*. *benthamiana*, and knocking-out the ortholog of *NbMIP1* family in rice also weaken the viral infection. Our study reveals the dual roles of the UPR in viral infection, and exemplifies an arms race between the plant and RSV.

## Results

### RSV infection induces host UPR and activates the autophagy pathway

**Fig 1:**
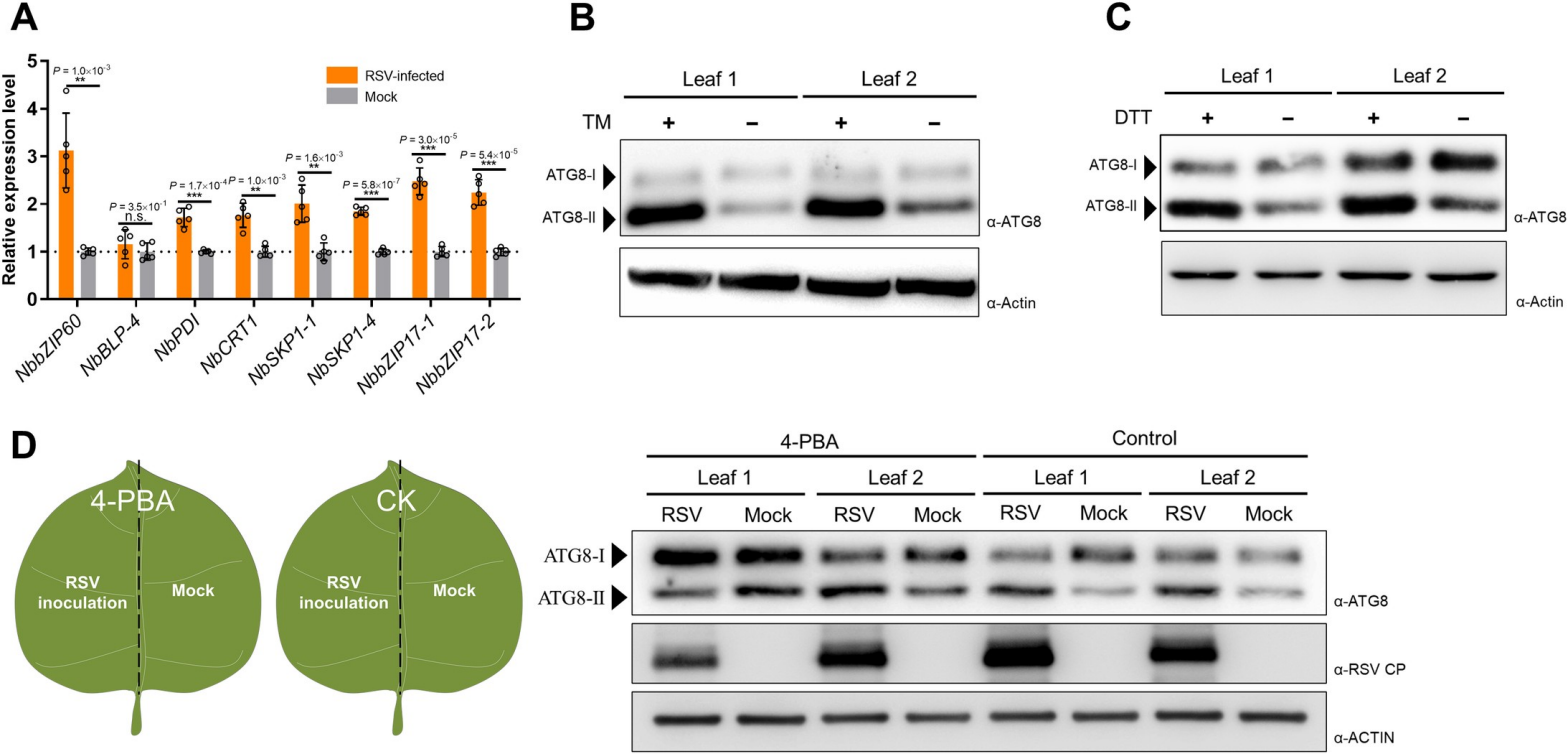
RSV infection induces host UPR and activates the autophagy pathway. **(A)** RT-qPCR analysis of the UPR-related genes in RSV-infected or healthy *N*. *benthamiana* at 12 dpi. *NbActin* was used as an internal reference in relative quantification. The values represent the means of relative expression levels ± SD relative to the mock plants (*n* = 4 biological replicates). Data were analyzed by Student’s *t*-test, and asterisks denote significant differences between RSV-infected and mock plants (two-sided, **P < 0.01, ***P < 0.001, n.s., not significant). **(B and C)** Western blotting analysis of NbATG8 in *N*. *benthamiana* leaves treated with TM (B) or DTT (C). Each half of a leaf was treated with TM or DMSO for the TM test (B), DTT or ddH_2_O for the DTT test (C). The total protein was extracted at 18 hpt following by western blotting analysis. NbATG8 were detected by specific antibodies. Actin was used as loading controls. **(D)** Western blotting analysis of NbATG8 in RSV inoculated leaves pre-treated with 4-PBA. *N*. *benthamiana* leaves were pre-treated with 4-PBA at 12 h before inoculating RSV. The leaves were harvested at 6 dpi, and total protein was extracted for western blotting. Actin was used as loading controls. RSV CP indicated virus infection.

In order to investigate whether RSV infection can induce the UPR in plant cells, RT-qPCR was performed to monitor the transcriptional changes of UPR-related genes which are commonly used as markers for the UPR and ER stress [15]. *NbPDI*, *NbCRT1*, *NbSKP1*, *NbbZIP17*, and *NbbZIP60* were significantly upregulated at 12 dpi (Fig 1A), indicating that RSV infection induces the UPR in plant cells.

Autophagy is often activated by ER stress to eliminate unfolded proteins [22,23]. Therefore, we suspected that the host’s autophagy pathway could also activated by the RSV-induced UPR. We first confirmed that RSV infection activated the host autophagy pathway. RSV-infected systemic leaves were harvested at 12 dpi, and the level of the NbATG8 protein during the viral infection was assessed by using Western blotting. As shown, NbATG8 accumulation in infected tissues was higher than that in non-inoculated controls (S1A Fig). Accumulation of NbATG8-II, an indicator of activated autophagy pathway, was also detected in infected samples, indicating a higher level of autophagic flux in RSV-infected *N*. *benthamiana* (S1A Fig). To determine the facticity of the bands which we considered NbATG8-II, we silenced *NbATG3*, *NbATG5*, and *NbATG7*, the genes involved in ATG8 processing, and *NbTOR*, a critical negative regulator in the autophagy pathway, by using tobacco rattle virus (TRV)-based virus-induced gene silencing vector. The silencing efficiency of these genes was confirmed by RT-qPCR (S2A Fig). As expected, bands of NbATG8-II disappeared when silencing *NbATG3*, *NbATG5*, and *NbATG7*, and accumulated further in *NbTOR*-silenced plants, indicating a high level of autophagic flux (S3 Fig).

To address whether there are more autophagosomes in RSV-infected cells, RFP-NbATG8f1 was expressed in RSV systemically infected or healthy plant leaves at 12 dpi. As shown, more punctate structures containing RFP-NbATG8f1 were observed in RSV-infected leaves (S1B Fig). The number of these structures per cell was calculated, and statistical analysis was performed, demonstrating that there were significantly more autophagosome-like structures in RSV-infected cells (S1C Fig). Transmission electron microscopy (TEM) was also employed to analyze the cell ultrastructure of RSV-infected plants, which further confirmed the increased number of autophagosome-like structures both in RSV-inoculated and systemically infected leaves (S1D and S1E Fig).

Next, we asked whether RSV-induced UPR was directly related to the activation of the autophagy pathway. Dithiothreitol (DTT) and tunicamycin (TM) are chemicals used to trigger ER stress and activate the UPR [12]. To test ER stress and the UPR can activate the autophagy in *N*. *benthamiana*, 2mM DTT or 5 μg/mL TM was infiltrated into *N*. *benthamiana* leaves. A significant increase of NbATG8-II after treatment of TM or DTT was observed (Fig 1B and 1C). To further explore the delicate relationship between UPR and RSV infection, 4-phenylbutyric acid (4-PBA), a chemical chaperone that reduces ER stress and inhibits the UPR [12,39], was used to treat leaves before inoculating RSV. To compare the effects of 4-PBA on autophagic flux levels in parallel, each half of a leaf was rubbed with the sap of RSV-infected or healthy rice. Western blotting analysis showed that the increased accumulation of NbATG8-II was inhibited after treatment with 4-PBA (Fig 1D), indicating that the activated autophagy after RSV infection is associated with ER stress-related UPR.

### RSV-encoded NSvc4 is targeted for autophagic degradation

**Fig 2:**
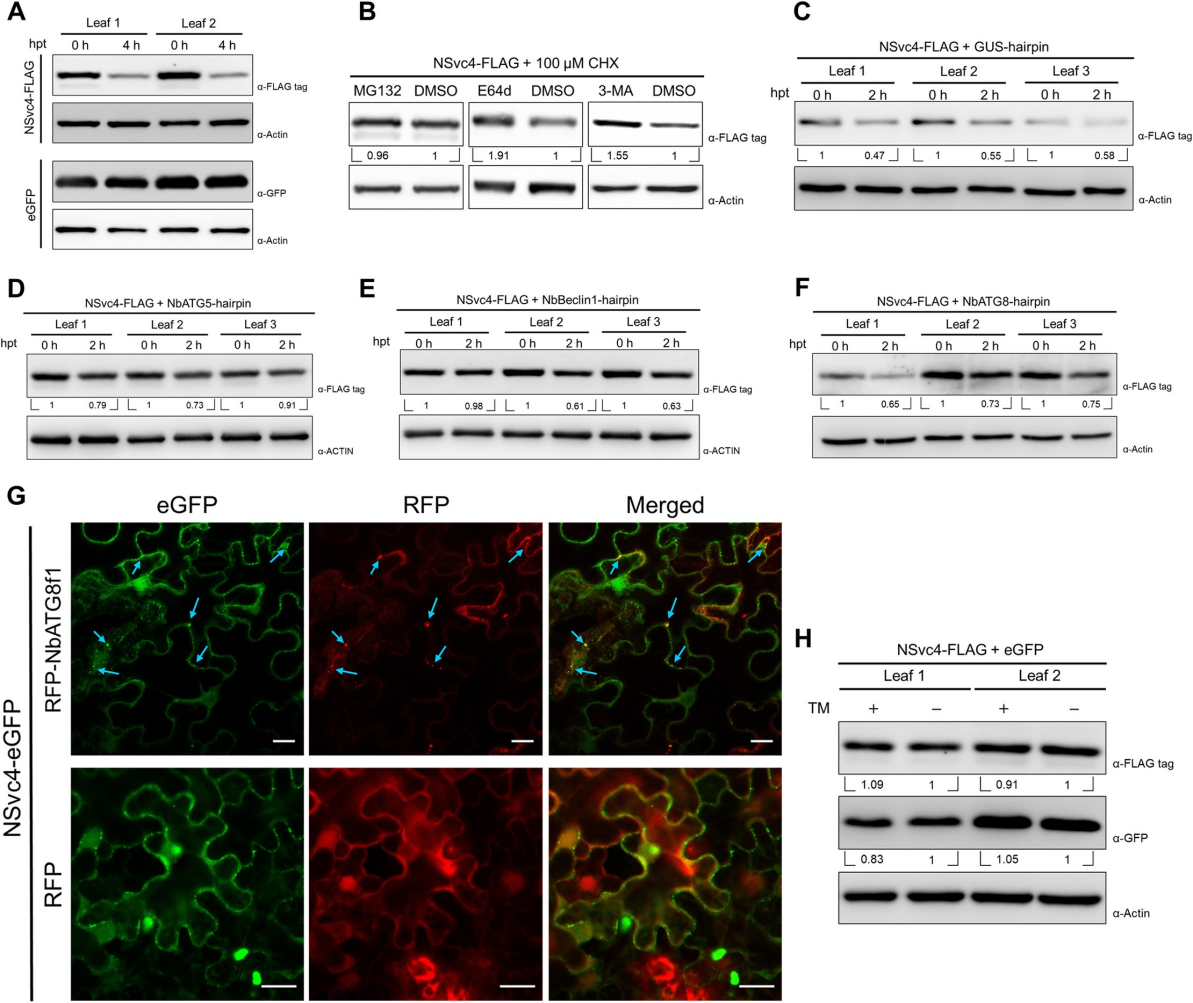
NSvc4 is unstable in plant cells and degraded through the autophagy pathway. **(A)** *In vivo* protein stability assay of NSvc4. The leaves expressing NSvc4-FLAG or eGFP were treated with 100 μM CHX. Samples were harvested at 0 h and 4 h after treatment for western blotting. Actin was used as a loading control. **(B)** Protease inhibitors treatment of the leaves expressing NSvc4-FLAG. The leaves expressing NSvc4-FLAG were treated with 100 μM CHX together with MG132, E64d, 3MA, or DMSO, respectively. Six hours later, the leaves were harvested, and total protein was extracted for western blotting. Actin was used as a loading control. The relative levels of NSvc4-FLAG to Actin were calculated and normalized by setting values in DMSO treatment as 1.0. **(C-F)** *In vivo* protein degradation rate assay of NSvc4 upon silencing *NbATG5*, *NbBeclin1* or *NbATG8*. The leaves expressing NSvc4-FLAG with GUS-hairpin (C), NbATG5-hairpin (D), NbBeclin1-hairpin (E) or NbATG8-hairpin (F) were treated with CHX. Samples were harvested at 0 h and 2 h after treatment for western blotting. Actin was used as a loading control. The relative levels of NSvc4-FLAG to Actin were calculated and normalized by setting values at 0 h as 1.0. **(G)** Confocal images of NSvc4-eGFP co-expressed with RFP-NbATG8f1. *N*. *benthamiana* leaves were infiltrated with agrobacteria carrying NSvc4-eGFP and RFP-NbATG8f1 or RFP. Arrows indicate colocalized eGFP and RFP fluorescence. Bars, 20 μm. (**H**) Western blotting analysis of NSvc4-FLAG after TM treatment in *N*. *benthamiana* leaves. NSvc4-FLAG and eGFP were co-expressed in *N*. *benthamiana* leaves by agroinfiltration. Then, the leaves were treated with TM at 42 hpi. Eighteen hours later, total protein was extracted for western blotting analysis. The relative levels of NSvc4-FLAG to Actin were calculated and normalized by setting values in DMSO control as 1.0.

Plant autophagy pathway has been reported to negatively regulate the infection of RSV, and silencing the autophagy-related genes in *N*. *benthamiana* promotes the infection of RSV [27]. We suspect the UPR-activated autophagy might promote viral protein(s) to be degraded. NSvc4, the movement protein of RSV, is crucial for RSV movement and infection [5,7,9]. In our experiment, we have consistently observed that NSvc4 protein expression is not stable when transiently expressed in plant cells. To validate this and further investigate the potential pathways involved in NSvc4 degradation, we analyzed the stability of NSvc4 *in vivo* by using cycloheximide (CHX), a protein synthesis inhibitor, to treat *N*. *benthamiana* leaves expressing NSvc4-FLAG. After CHX treatment, the synthesis of NSvc4 is blocked, while protein degradation continues. As shown in Fig 2A, the accumulation of NSvc4-FLAG decreased significantly at 4 h after CHX treatment, while GFP, which is considered a relatively stable protein [25], displayed no apparent changes, confirming that NSvc4 is unstable in plant cells.

There are two main protein degradation pathways in eukaryotes: the ubiquitin-proteasome pathway and the autophagy pathway. To elucidate how NSvc4 is degraded, chemical inhibitors of the two pathways were used. Plant leaves expressing NSvc4-FLAG were treated with MG132, an inhibitor of the proteasome, or E64d and 3-MA, inhibitors of autophagy. As shown in Fig 2B, the accumulation of NSvc4-FLAG was notably higher in samples treated with autophagy inhibitors (E64d and 3-MA). However, no significant difference was observed in the following treatment with MG132. Next, we monitored the NSvc4 degradation rate *in vivo*. Leaves expressing NSvc4-FLAG were treated with CHX, then samples were collected at 0 and 2 hours post-treatment (hpt). When co-expressed with a hairpin against GUS (as control), the NSvc4-FLAG protein level was much lower at 2 hpt (Fig 2C); in contrast, co-expression with hairpins against *NbATG5*, *NbBeclin1* or *NbATG8*, the key components of the autophagy pathway, significantly raised NSvc4-FLAG protein levels at 2 hpt (Fig 2D–2F). The silencing efficiency of these hairpins was determined by qRT-PCR (S2B and S2C Fig). To address whether NSvc4 could be transported to autophagosomes, NSvc4-eGFP was co-expressed with RFP-NbATG8f1 or RFP in *N*. *benthamiana* leaves. We found that NSvc4-eGFP and RFP-NbATG8f1 co-localized in punctate structures, while no co-localization of NSvc4-eGFP and RFP was observed (Fig 2G). Taken together, these results suggest that NSvc4 is delivered to autophagosomes and degraded through the canonical autophagy pathway.

We next asked whether ER stress-induced autophagy could promote the degradation of NSvc4 directly. The *N*. *benthamiana* leaves expressing NSvc4-FLAG and eGFP were treated with TM. Then, the total protein was extracted for western blotting analysis. As expected, the TM treatment did not have obvious effects on the protein level of eGFP. Unexpectedly, the protein level of NSvc4-FLAG did not decrease significantly after TM treatment (Fig 2H), indicating that there might be some host factors involved in fine-tuning the stability of NSvc4 in plant cells during ER stress and protecting NSvc4 from ER stress-induced autophagic degradation.

### NSvc4 interacts with UPR-elicited type-I J-domain proteins, NbMIP1s

To identify host proteins participating in the regulation of NSvc4 stability, NSvc4 was cloned into the pGBDT7 vector and used as the bait to screen a yeast cDNA library of *N*. *benthamiana*. A host type-I J-domain protein was found to interact with NSvc4 in yeast cells. A BLAST search shows that this protein belongs to the NbMIP1s family [38]. InterPro scanning indicates that the members of the NbMIP1 family contain highly conserved domains of type-I DNAJ family protein (S4A Fig). Protein sequence alignment with all other proteins in the NbMIP1 family shows that the protein identified in the yeast two-hybrid screen shares the highest similarity (96.9% identity) with NbMIP1.2. Based on the protein sequence analysis, the gene encoding this protein was named *NbMIP1*.*2b*, and the homologous gene reported by Du *et al*. was designated as *NbMIP1*.*2a* (S4B Fig) [38].

**Fig 3:**
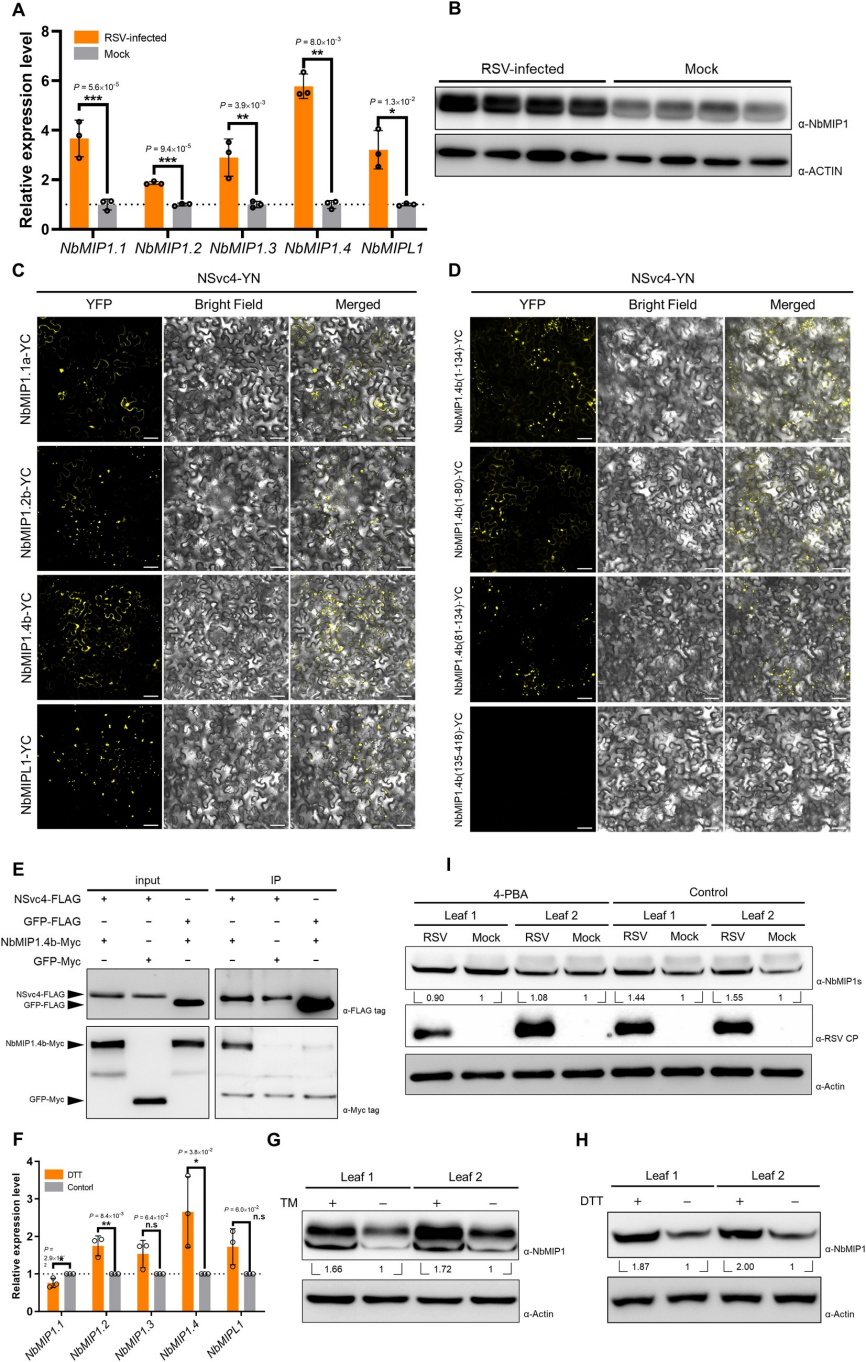
NbMIP1 family proteins are upregulated by RSV-induced UPR and interact with NSvc4. **(A)** RT-qPCR analysis of *NbMIP1* family expression level in RSV-infected or healthy *N*. *benthamiana*. *NbActin* was used as an internal reference in relative quantification. The values represent the means of the expression levels ± standard deviations (SD) relative to the mock plants (*n* = 3 biological replicates). Student’s *t*-test was performed, and asterisks denote significant differences between RSV-infected and mock plants (two-sided, *P < 0.05, **P < 0.01, ***P < 0.001). **(B)** Western blotting analysis of NbMIP1 family proteins in RSV-infected and mock plants at 12 dpi. Total protein was extracted for western blotting. Actin was used as a loading control. **(C and D)** BiFC assay of NSvc4 with NbMIP1.4b and its truncated versions. Leaves were infiltrated with agrobacteria carrying NSvc4 fused to the N-terminal part of YFP, and NbMIP1.4b or its truncated versions were fused to the C-terminal part of YFP. Samples were observed by laser confocal microscopy at 48 hpi. Bars, 50 μm. **(E)** Co-immunoprecipitation assay of NSvc4 with NbMIP1.4b. NSvc4-FLAG or GFP-FLAG were co-expressed with NbMIP1.4b-Myc or GFP-Myc in *N*. *benthamiana* leaves. 48 hours later, the leaf protein extracts were incubated with FLAG magnetic beads. Samples before (input) and after immunoprecipitation (IP) were analyzed by western blotting using anti-FLAG or anti-Myc antibodies. **(F)** RT-qPCR analysis of *NbMIP1*s expression levels after DTT treatment. Each half of a leaf was treated with DTT or ddH_2_O, respectively. The total RNA was extracted at 18 hpt. *NbActin* was used as an internal reference in relative quantification. The relative expression levels were normalized by setting the value of control as 1.0 in each leaf (*n* = 3 biological replicates). Then, data were analyzed by Student’s *t*-test, and asterisks denote significant differences between DTT-treated and control halves (two-sided, *P < 0.05, **P < 0.01, n.s., not significant). (**G and H**) Western blotting analysis of NbMIP1.4b in *N*. *benthamiana* leaves treated with TM (G) or DTT (H). The samples in Fig 1B and 1C were used for western blotting to detect NbMIP1s. The relative levels of NbMIP1s to Actin were calculated and normalized by setting values in control halves as 1.0. **(I)** Samples in Fig 1D were analyzed by western blotting using antibody against NbMIP1s. Actin was served as loading controls. RSV CP indicated virus infection.

To investigate whether the expression of *NbMIP1* family genes responds to RSV infection, RT-qPCR was conducted by using primers specific to the different members in this family. Our results show that the mRNA accumulation of all *NbMIP1* family genes was significantly upregulated during the viral infection (Fig 3A). Among them, NbMIP1.4 was upregulated nearly 6 folds, suggesting that NbMIP1.4 might play a major role in the response of *N*. *benthamiana* to RSV infection. There are two homologous genes in the *NbMIP1*.*4* subgroup, *NbMIP1*.*4a* and *NbMIP1*.*4b* (S4B Fig). Considering that *N*. *benthamiana* is allotetraploid, we postulate that *NbMIP1*.*4a* and *NbMIP1*.*4b* are alleles from two ancestries. Western blot also confirmed the upregulation of NbMIP1 at the protein level (Fig 3B). It is noteworthy that NbMIP1 family proteins show high similarity, and therefore the antibody against NbMIP1.4b would also detect other homologs of the family. Based on these findings, NbMIP1.4b was cloned as a representative member of the NbMIP1 family in subsequent experiments to study the function of NbMIP1 proteins.

To study the subcellular localization of the interaction between NbMIP1 and NSvc4, bimolecular fluorescence complementation (BiFC) assays were performed. *NbMIP1*.*1a*, *NbMIP1*.*2b*, *NbMIP1*.*4b*, and *NbMIPL1* were individually cloned and fused to the C-terminal part of the YFP, while the N-terminal part of the YFP was fused to NSvc4. As shown in Fig 3C, YFP fluorescence was observed in all these four combinations, indicating that all tested NbMIP1s can interact with NSvc4 *in vivo*. To identify the domain of NbMIP1 involved in its interaction with NSvc4, NbMIP1.4b truncated versions based on the predicted domains were constructed and tested. BiFC assays showed that 1–134 aa, 1–80 aa, and 81–134 aa of NbMIP1.4b interacted with NSvc4, while 135–418 aa did not (Fig 3D). Interestingly, the interaction of NbMIP1.4b (1–134 aa) and NbMIP1.4b (1–80 aa) with NSvc4 showed similar patterns to that of the full-length NbMIP1.4b, which was mainly distributed in the cytoplasm with some punctate structures. However, the interaction of NbMIP1.4b (81–134 aa) with NSvc4 was observed mostly in punctate structures. These results demonstrate that the N-terminal part of NbMIP1.4b, which mainly contains the J and the G/F domains, interacts with NSvc4. The interaction between NbMIP1 and NSvc4 was further confirmed by co-immunoprecipitation (Co-IP) assay, and western blotting of immunoprecipitated proteins show that NbMIP1.4b-Myc specifically co-precipitated with NSvc4-FLAG (Fig 3E).

The UPR also includes the increased expression levels of molecular chaperones. Therefore, we speculate that the RSV-induced UPR is also responsible for the upregulation of *NbMIP1*s. To confirm whether ER stress and the UPR could induce the upregulation of *NbMIP1*s, *N*. *benthamiana* leaves were treated by DTT or TM. At 24 hpt, RT-qPCR showed that the *NbMIP1*s transcript accumulation increased after DTT treatment (Fig 3F). Western blotting also showed that the NbMIP1s protein level increased after TM or DTT treatment (Fig 3G and 3H). Notably, *NbMIP1*.*4* was the most highly upregulated gene among the tested NbMIP1 family genes, which is consistent with the gene expression pattern of *N*. *benthamiana* in response to the RSV infection. We also compared the effects of 4-PBA on NbMIP1s expression upon RSV infection. The western blotting analysis of samples in Fig 1D showed that the increased accumulation of NbMIP1s was inhibited after treatment with 4-PBA (Fig 3I). Our results indicate that RSV-induced UPR upregulates the expression levels of *NbMIP1*s.

### NbMIP1.4b stabilizes NSvc4 from autophagic degradation in an HSP70 chaperone-independent manner

**Fig 4:**
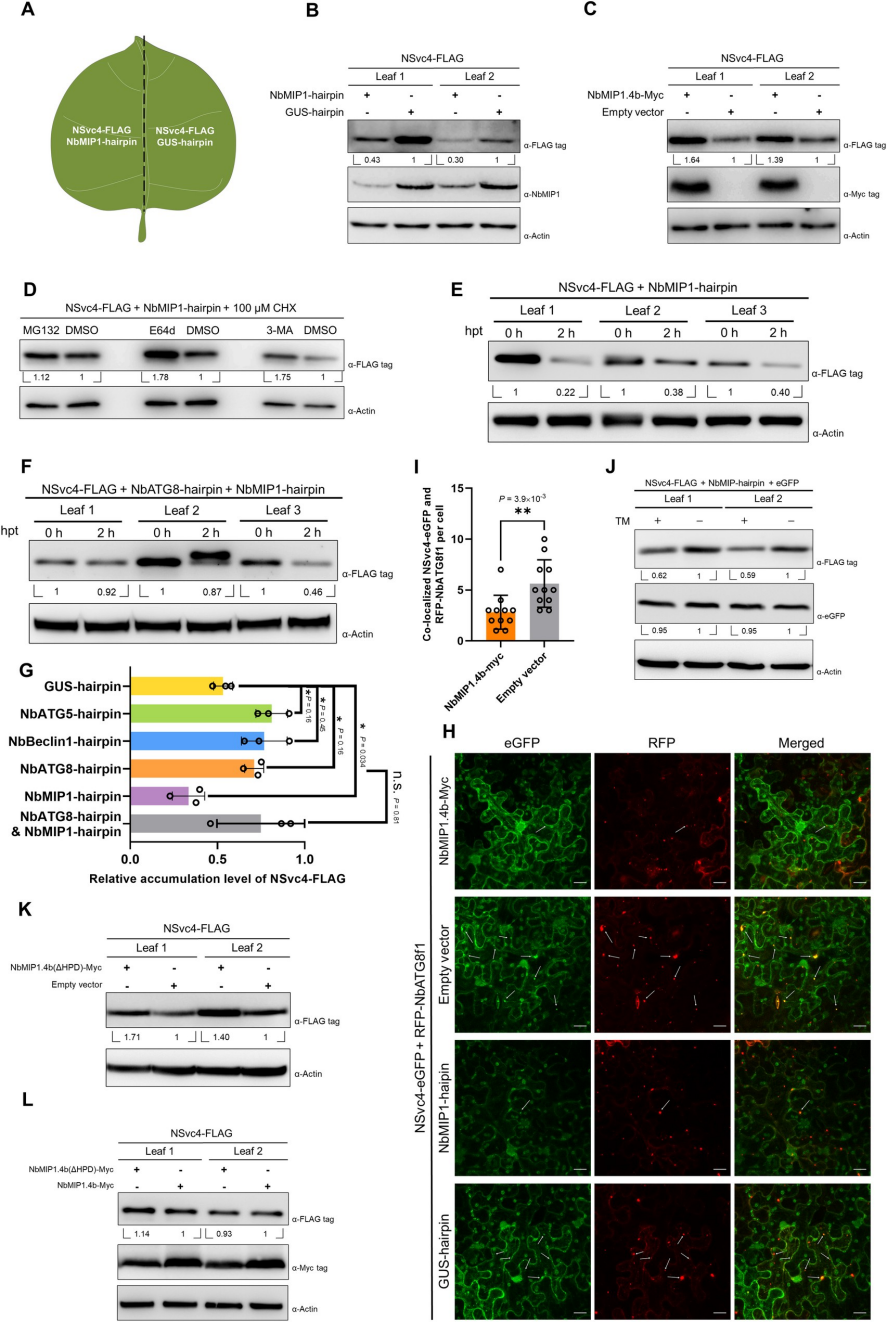
NbMIP1.4b stabilizes NSvc4 in an HSP70 chaperone-independent manner, and silencing NbMIP1s promotes NSvc4 degradation. **(A, B and D)** Silencing NbMIP1s impairs the stability of NSvc4. NSvc4-FLAG was co-expressed with NbMIP1-hairpin or GUS-hairpin in *N*. *benthamiana* leaves. Total protein was extracted for western blotting at 60 hpi. The polyclonal antibody against NbMIP1s was used to detect NbMIP1s protein level. The relative levels of NSvc4-FLAG to Actin were calculated and normalized by setting the values in GUS-hairpin treatment as 1.0. Meanwhile, some of the leaves were treated with MG132, E64d, 3MA, and DMSO, and then, these leaves were harvested at 6 hpt followed by western blotting. The relative levels of NSvc4-FLAG to Actin were normalized by setting the values in DMSO treatment as 1.0. **(C)** Expressing NbMIP1.4b stabilized NSvc4. NSvc4-FLAG was co-expressed with NbMIP1.4b-Myc or empty vector in *N*. *benthamiana* leaves. Samples were harvested at 60 hpi for western blotting. The expression of NbMIP1.4b-Myc was confirmed by the anti-Myc antibody. The relative levels of NSvc4-FLAG to Actin were normalized by setting the values in empty vector control as 1.0. **(E and F)** *In vivo* protein degradation rate assay of NSvc4 upon silencing NbMIP1s. The leaves expressing NSvc4-FLAG with NbMIP1-hairpin or NbMIP1-hairpin and NbATG8-hairpin were treated with CHX. The samples were harvested at 0 h and 2 h after treatment. Total protein was extracted for western blotting. The relative levels of NSvc4-FLAG to Actin were normalized by setting the values at 0 h as 1.0. **(G)** The relative levels of NSvc4-FLAG from Fig 2C–2F, and Fig 4E and 4F, were used to plot a histogram. Student’s *t*-test was performed, and asterisks denote significant differences between different treatments (two-sided, *P < 0.05, n.s., not significant). **(H)** Confocal images of NSvc4-eGFP and RFP-NbATG8f1 co-expressed with NbMIP1.4b-Myc or NbMIP1-hairpin. NSvc4-eGFP and RFP-NbATG8f1 were co-expressed with NbMIP1.4b, empty vector, NbMIP1-hairpin, or GUS-hairpin in *N*. *benthamiana* leaves by agroinfiltration, respectively. Then, the leaves were observed by confocal microscope at 60 hpi. Arrows indicate the NSvc4-eGFP and RFP-NbATG8f1 co-localized punctate structures. Bars, 20 μm. **(I)** Numbers of NSvc4-eGFP and RFP-NbATG8f1 co-localized punctate spots in NbMIP1.4b-Myc or empty vector-expressed leaves were calculated per cell and analyzed by student’s *t*-test (two-sided, **P < 0.01). (**J**) Western blotting analysis of NSvc4-FLAG after TM treatment in *N*. *benthamiana* leaves upon silencing *NbMIP1*s. NSvc4-FLAG, eGFP and NbMIP1-hairpin were co-expressed in *N*. *benthamiana* leaves by agroinfiltration. Then, the leaves were treated with TM at 42 hpi. Eighteen hours later, total protein was extracted for western blotting analysis. The relative levels of NSvc4-FLAG and eGFP to Actin were calculated and normalized by setting values in DMSO control as 1.0. **(K and L)** NbMIP1.4b(ΔHPD) mutant stabilizes NSvc4. The experiment is similar to (4C).

DnaJ proteins are known to involve in regulating protein stability primarily by stimulating the ATPase activity of chaperone proteins, HSP70s. To investigate the possible roles of NbMIP1s playing in regulating NSvc4 stability, first, NbMIP1s were silenced by expressing a hairpin targeting the whole NbMIP1 gene family. The NbMIP1-hairpin or a GUS-hairpin as a control was co-expressed with NSvc4-FLAG in *N*. *benthamiana* in each half of the same leaf (Fig 4A). Total proteins were extracted at 60 hpi, followed by western blotting. Our results show that the protein level of NSvc4-FLAG co-expressed with NbMIP1-hairpin was significantly lower than that co-expressed with the GUS-hairpin control, suggesting that the protein stability is reduced after silencing *NbMIP1*s (Fig 4B). The silencing efficiency of the NbMIP1-hairpin was determined by western blotting analysis of NbMIP1s (Fig 4B). Next, NbMIP1.4b-Myc was co-expressed with NSvc4-FLAG or empty vector control. As shown in Fig 4C, the accumulation of NSvc4-FLAG was almost 1.5-fold higher when co-expressed with NbMIP1.4b-Myc.

Then we asked whether the degradation of NSvc4 caused by silencing *NbMIP1*s was also related to the autophagy pathway. The use of the autophagy inhibitors E64d and 3-MA could reduce the effect of silencing *NbMIP1*s on NSvc4 accumulation, indicating that the degradation of NSvc4 caused by this silencing is also due to autophagy (Fig 4D). Moreover, silencing of *NbMIP1*s sped up NSvc4-FLAG degradation, while the effect was lost when concomitantly silencing *NbATG8* (Fig 4E–4G). The silencing efficiency of the NbATG8-hairpin and the NbMIP1-hairpin in this experiment was confirmed by RT-qPCR or western blotting (S2C and S2D Fig). Then we co-expressed NSvc4-eGFP and RFP-NbATG8f1 with NbMIP1.4b-Myc or NbMIP1-hairpin in *N*. *benthamiana* leaves, and then the co-localization of NSvc4-eGFP and RFP-NbATG8f1 was determined by confocal microscope. We found that the number of eGFP and RFP co-localized punctate structures in NbMIP1.4b-Myc expressed leaves was significantly lower than that in empty vector control (Fig 4H and 4I), suggesting NbMIP1s might stabilize NSvc4 by prevent it entering the autophagosome. However, in *NbMIP1*s-silenced leaves, the fluorescence intensity of NSvc4-eGFP decreased significantly, and surprisingly, only few eGFP and RFP co-localized punctate structures were observed (Fig 4H). We speculate that silencing *NbMIP1*s will promote NSvc4 to be transported to the autophagosome and degraded rapidly, which dramatically reduce the accumulation level of NSvc4-eGFP. Thus, NSvc4-eGFP was barely observed co-localized with autophagosome-like structures in *NbMIP1*s-silenced leaves.

In the above results, we found that TM treatment could not promote the degradation of NSvc4, and we suspected there might be host factors which protected NSvc4 from the ER stress-induced autophagic degradation. To confirm whether NbMIP1s are the factors, TM was used to treat the *N*. *benthamiana* leaves expressing NSvc4-FLAG and eGFP upon silencing *NbMIP1*s by hairpin. We found that TM treatment prominently impaired the stability of NSvc4-FLAG, while eGFP remained stable (Fig 4J), which indicates that the ER stress-induced autophagy can only promote the degradation of NSvc4 upon silencing NbMIP1s. When the autophagy is activated by the UPR, the NbMIP1s, which are also upregulated by the UPR, can be utilized by NSvc4 to evade the autophagy-mediated degradation.

Our results above show that NbMIP1.4b interacts with NSvc4 not through its zinc finger-CTD domain but through its N-terminal J and G/F domains (Fig 3D), which conflicts with the canonical model of HSP40-HSP70 complex formation [32]. To investigate whether NbMIP1s regulate NSvc4 stability in a canonical HSP70 chaperone-dependent manner or not, an HPD-deleted NbMIP1.4b mutant [NbMIP1.4b(ΔHPD)] was generated and co-expressed with NSvc4-FLAG. Previous studies have shown that mutation of the HPD motif in the J domain dramatically impairs the ability of HSP40 to act as a co-chaperone to stimulate the ATPase activity of the HSP70 protein [32,34–36,40]. As shown in Fig 4K, an increased NSvc4 accumulation was observed when the protein was co-expressed with NbMIP1.4b(ΔHPD), and there was no significant difference between co-expression with this deleted version or the wild type (Fig 4L). The interaction between NbMIP1.4b(ΔHPD) and NSvc4 was confirmed by BiFC (S5 Fig). Therefore, our findings suggest that NbMIP1.4b stabilizes NSvc4 from autophagic degradation independently of the HSP70 chaperone network.

### Type-I J-domain NbMIP1 proteins promote RSV infection

**Fig 5:**
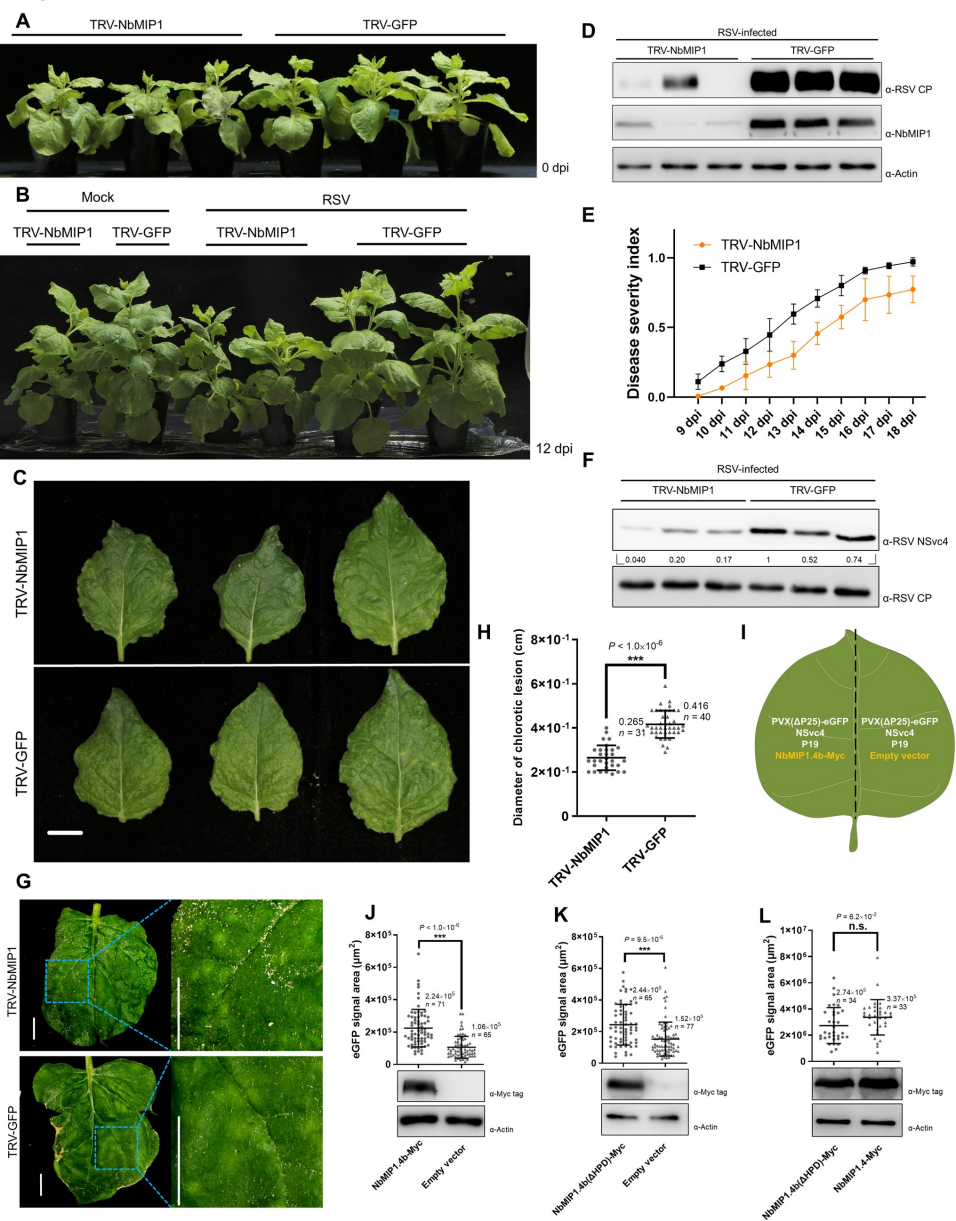
Silencing NbMIP1s delays RSV infection, and expressing NbMIP1.4b enhances the ability of NSvc4 to rescue the cell-to-cell movement of PVX(Δp25)-eGFP. **(A)** The phenotype of silencing NbMIP1s by TRV-based VIGS. The image was taken at 10 days after inoculation of TRV. **(B and C)** The symptom of TRV-NbMIP1 and TRV-GFP pre-inoculated plants at 12 dpi of RSV. Bars, 1 cm. **(D)** Time-course analysis of disease severity index (DSI). The experiments were carried out 3 times independently. Value at each point represents the mean of DSI ± SD. **(E)** Detection of RSV CP accumulation in TRV-NbMIP1 and TRV-GFP pre-inoculated plants. The leaves from RSV-infected plans in (C) were harvested. Total protein was extracted for western blotting. The protein level of RSV CP represented virus accumulation level, and the protein level of NbMIP1s indicated silencing efficiency. **(F)** Detection of relative protein level of NSvc4 in TRV-NbMIP1 and TRV-GFP pre-inoculated plants. The total protein of the leaves showed similar symptom severity were extracted for western blotting. The relative levels of NSvc4 to RSV CP were calculated and normalized by setting the value of the first lane of TRV-GFP plant as 1.0. **(G and H)** Measurements of the chlorotic lesion diameter on local leaves. Images of local leaves from TRV-NbMIP1 and TRV-GFP pre-inoculated plants were taken at 11 dpi. Areas of leaves were magnified to show the detail of chlorotic lesions. Bars, 1.0 cm. The diameter of these lesions was measured and analyzed by student’s *t*-test (two-sided, ***P < 0.001); the mean and the number of measured lesions (*n*) were labeled. **(I-L)** Analysis of the effects of NbMIP1.4b and HPD-motif-deleted mutant to the NSvc4 rescued cell-to-cell movement of PVX(Δp25)-eGFP. *Agrobacterium* carrying NbMIP1.4b-Myc, or NbMIP1.4b(ΔHPD)-Myc, or empty vector was mixed with agrobacteria carrying 800-fold-diluted PVX(Δp25)-eGFP, NSvc4, and TBSV p19. Each mixture was infiltrated into each half of the same leaf. The leaves were observed under fluorescence stereoscopic microscopy at 5 dpi. The area of fluorescence was measured by ZEN lite software and analyzed by student’s *t*-test (two-sided, *** *P* < 0.001, n.s., not significant); the mean and the number of measured area (*n*) were labeled. Meanwhile, leaves were harvested for western blotting to confirm the expression of NbMIP1.4b-Myc or its mutant.

Next, we asked whether the NbMIP1s played a significant role in the RSV infection. We silenced the *NbMIP1* family systemically by using TRV-based VIGS and challenged the silenced plants with RSV. *NbMIP1*s-silenced plants showed slightly reduced height compared with the plants infected with the TRV-GFP control (Fig 5A). Following RSV infection, noticeable vein yellowing was observed in the TRV-GFP control plants, while this symptom was milder in the TRV-NbMIP1 plants at 12 dpi (Fig 5C). Western blotting showed that the accumulation of the RSV coat protein (CP) in *NbMIP1*s-silenced plants was significantly lower than that in TRV-GFP-infected plants, correlating with viral symptoms (Fig 5D). To monitor the viral infection, a disease index was calculated based on the severity of the symptoms. As shown in Fig 5E, viral symptoms developed more slowly in *NbMIP1*s-silenced plants. At 20 dpi, TRV-GFP-infected plants displayed a more severe leaf curling symptom (S6A Fig). Notably, although the plant height of RSV-infected *NbMIP1*s-silenced plants remained lower at 12 dpi (Fig 5B), it surpassed that of RSV-infected control plants at 28 dpi (S6B Fig). These findings demonstrate that silencing of *NbMIP1*s delays RSV infection and symptom development.

To test whether silencing of *NbMIP1*s would reduce the NSvc4 protein level in the context of the viral infection, we determined the relative NSvc4 to CP protein level. Since silencing of *NbMIP1*s delays the RSV infection, while the levels of viral proteins may vary at different stages of the infection, RSV-infected leaves displaying similar symptoms were collected at different time points from *NbMIP1*s-silenced and TRV-GFP-infected control plants. RSV CP was used as a loading control. The NSvc4 accumulation in *NbMIP1*s-silenced plants was significantly lower than that in control plants (Fig 5F). Our group has previously shown that RSV infection causes chlorotic lesions on inoculated leaves, which are closely correlated with virus spreading [9]. To determine if the degradation of NSvc4 caused by silencing of *NbMIP1*s could inhibit the cell-to-cell movement of RSV, the diameter of chlorotic lesions was measured and analyzed. The lesion area on the leaves of NbMIP1s-silenced plants was almost half the size of that on control leaves on average (Fig 5G and 5H). These results illustrate that silencing *NbMIP1*s inhibits RSV infection by impairing the stability of NSvc4, resulting in a restriction of the virus cell-to-cell movement.

Previous research has found that NSvc4 can rescue the cell-to-cell movement of a movement protein-deficient mutant of potato virus X (PVX) [5,9]. To determine whether NbMIP1.4b expression could enhance the virus cell-to-cell movement mediated by NSvc4, we co-expressed NSvc4 and the movement protein-deficient PVX(Δp25)-eGFP with NbMIP1.4b-Myc or the empty vector on each half of a single leaf (Fig 5I). As expected, expressing NbMIP1.4b did promote PVX(Δp25)-eGFP spread. The eGFP-expressing area was significantly larger when NbMIP1.4b was co-expressed (Fig 5J). Since NbMIP1.4b stabilizes NSvc4 in an HSP70-independent manner, we speculated that the NbMIP1.4b co-chaperone-deficient mutant NbMIP1.4b(ΔHPD) might retain the capability to promote viral spread. As expected, NbMIP1.4b(ΔHPD) increased the virus spreading area similarly to wild-type NbMIP1.4b (Fig 5K and 5L). To rule out the possibility that NbMIP1.4b promotes PVX movement by enhancing its replication or protein expression, PVX(Δp25)-eGFP and tomato bushy stunt virus p19 were co-expressed with NbMIP1.4b-Myc or empty vector. The protein levels of PVX CP and eGFP were examined by western blot, and the intensity of eGFP fluorescence was evaluated by using a hand-held UV lamp. No significant differences in eGFP fluorescence were observed when NbMIP1.4b-Myc was co-expressed, indicating that NbMIP1.4b cannot enhance PVX replication or protein expression (S7 Fig).

### The rice homologue of NbMIP1.4, OsDjA5, plays a positive role in RSV infection

**Fig 6:**
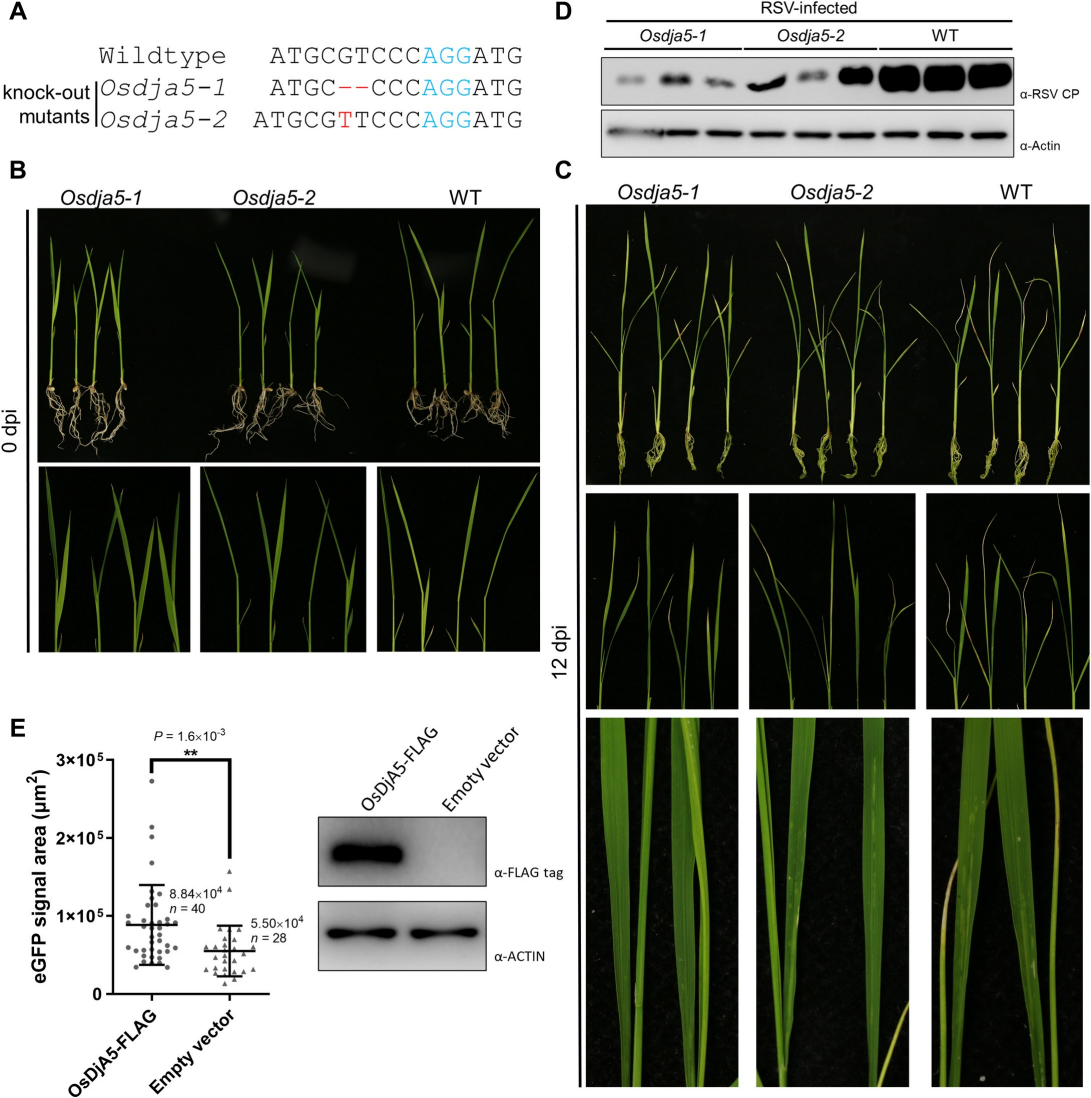
Challenge rice *Osdja5* knock-out mutants with RSV. **(A)** Diagram of frameshift mutations in *Osdja5-1* and *Osdja5-2*. The blue bases represent the PAM sequence, and red bases mark mutation sites. **(B)** The phenotype of *Osdja5* knock-out mutants. **(C and D)** The symptom (C) and virus accumulation levels (D) of *Osdja5* mutants and wild type plants infected by RSV at 12 dpi. The virus accumulation levels were detected by western blotting using a monoclonal antibody against RSV CP and Actin was used as a loading control here. **(E)** Analysis of the effects of OsDjA5 to the NSvc4 rescued cell-to-cell movement of PVX(Δp25)-eGFP. *Agrobacterium* carrying OsDjA5- FLAG or empty vector was mixed with agrobacteria carrying 800-fold-diluted PVX(Δp25)-eGFP, NSvc4, and TBSV p19. Each mixture was infiltrated into each half of the same leaf. The leaves were observed under fluorescence stereoscopic microscopy at 5 dpi. The area of eGFP fluorescence was measured by ZEN lite software and analyzed by student’s *t*-test (two-sided, **P < 0.01); the mean and the number of measured area (*n*) were labeled. Western blotting confirmed the expression of OsDjA5-FLAG.

To find out whether the homologous genes of *NbMIP1* in rice, the original host of RSV, also affect RSV infection, a blastp search in the rice protein database (http://rice.plantbiology.msu.edu/) was performed by using the amino acid sequence of NbMIP1.4b as the query. A phylogenetic tree was generated [41], and four genes from rice clustered together with NbMIP1s (S8A Fig). The proteins encoded by these four genes interacted with NSvc4 *in vivo*, as verified by BiFC assay (S8B Fig). RT-qPCR confirmed the upregulation of these genes after RSV infection (S8C Fig). Interestingly, *OsDjA5*, which possesses the highest protein similarity to *NbMIP1*s, also showed the highest upregulation level among these four genes, suggesting a conserved function between *OsDjA5* and *NbMIP1* family in the RSV infection. Next, knock-out mutants of *OsDjA5* were generated by CRISPR/Cas9. The frameshift mutations in *Osdja5-1* and *Osdja5-2* were confirmed by Sanger sequencing (Fig 6A). Two independent mutant lines showed a mild dwarf phenotype (Fig 6B), similar to that observed in *NbMIP1*s-silenced *N*. *benthamiana* plants. Twelve days after challenge with RSV by using small brown planthopper-mediated inoculation, the mutant lines developed milder symptoms than WT-plants (Fig 6C), and lower virus accumulation levels were also detected in mutant lines (Fig 6D). A movement complementation assay also indicates that OsDjA5 can enhance the cell-to-cell movement of PVX(Δp25)-eGFP rescued by NSvc4 (Fig 6E).

## Discussion

The UPR plays important roles in the infection of plant viruses, while the effects of the UPR to plant negative-strand viruses are still unclear. In this study, we find the infection of RSV elicits the UPR in plant cells, which also activates the autophagy pathway. NSvc4 is the movement protein of RSV and is crucial for RSV infection. We provide evidence that NSvc4 is not stable upon ectopic expression and is degraded through the autophagy pathway. We then identify the UPR-upregulated type-I J-domain NbMIP1 proteins as interacting proteins of NSvc4 and demonstrate that they protect NSvc4 from autophagic degradation in an HSP70 chaperone-independent manner. Silencing *NbMIP1*s in *N*. *benthamiana* or knocking-out *OsDjA5* in rice delays RSV infection, while over-expressing *NbMIP1*.*4b* promotes virus cell-to-cell movement.

The ER is the main protein synthesis factory in the cell, which is required by the viruses to synthesize their proteins during the infection. Also, the replication and intracellular transport of some viruses involve the host ER endomembrane system [11]. Many plant viruses are capable of eliciting the UPR in host cells, and the UPR is also required by the viruses to infect host cells [12–16,18]. In general, the massive amount of virus-encoded proteins may not fold properly and accumulate in the ER, or some viral membrane-associated proteins may modify the structure of the ER membrane, which in turn affects ER homeostasis and activates the UPR. The UPR involves a series of cellular processes including upregulation of molecular chaperones and activation of autophagy to buffer the ER stress [10]. However, the roles of the UPR in plant negative-strand viruses infections are still largely unknown. In this study we confirm that the infection of RSV elicits the UPR in *N*. *benthamiana* leaves (Fig 1A).

Autophagy is one of the main protein turnover pathways in eukaryotic cells, and extensive studies have unveiled its important roles in plant-virus interactions [24–26,26,27,42–44]. Although in many studies, the autophagy pathway is activated by plant viruses, few of them describe the relationship between virus-induced UPR and autophagy. In this study, we also found that RSV infection activated the autophagy pathway, as shown by the increased accumulation of NbATG8-II (S1A Fig). In addition, activation of the autophagy pathway after RSV infection was also confirmed by confocal microscopy and TEM, which demonstrates that more autophagosome-like structures are found upon RSV infection (S1B–S1E Fig). The TM or DTT treatment can activate the autophagy pathway in *N*. *benthamiana* (Fig 1B and 1C). 4-PBA is a chemical molecular chaperone, which attenuates the UPR in animal and plant cells [12,39]. The autophagy activation after RSV infection was inhibited in leaves pre-treated with 4-PBA (Fig 1D), indicating that the UPR eliciting is essential for autophagy activation upon RSV infection.

In many cases, autophagy functions as a defense mechanism against plant viruses. The cauliflower mosaic virus (CaMV) P4 protein is targeted by the plant selective autophagy receptor NBR1 (neighbor of BRCA1 gene 1) and degraded through the receptor-mediated selective autophagy pathway [26]. Beclin1 interacts with turnip mosaic virus (TuMV) RdRp and redirects TuMV RdRp to autophagosomes for degradation [24]. The C1 protein of tomato leaf curl Yunnan virus (TLCYnV) is directly targeted by ATG8 and exported from the nucleus to the cytoplasm for degradation [25]. It seems likely that the degradation of viral proteins through the autophagy pathway is a common strategy for plants to defend themselves against viruses. The multifaceted roles of autophagy in the RSV-host interaction have been demonstrated in a few studies. NSvc4 is reported to increase the SEL by inhibiting the S-acylation of remorin and inducing its autophagic degradation [9]. Silencing *NbATG7* blocks plant autophagy and facilitates RSV infection, which indicates that autophagy plays an anti-RSV role in *N*. *benthamiana* [27]. In the present study, we found that NSvc4 was unstable in plant cells (Fig 2A). Then, the chemical inhibitors of protein degradation pathways were used to analyze NSvc4 degradation. Only inhibitors of autophagy increased the NSvc4 protein level (Fig 2B). In addition, silencing *NbATG8*, *NbATG5 or NbBeclin1*, the core components of the autophagy machinery, reduced the degradation rate of NSvc4 (Figs 2C–2F and 4G). Moreover, NSvc4-eGFP co-localized with RFP-NbATG8f1 in punctate autophagosome-like structures (Fig 2G). These pieces of evidence support the model proposing that NSvc4 is prone to be degraded through the canonical autophagy pathway. The exact mechanism underpinning the transport of NSvc4 to autophagosomes, and whether receptor-mediated selective autophagy pathway plays a role in this process requires further investigation. A previous study found a transmembrane domain in NSvc4, which is required for NSvc4 to associate with the plasma membrane (PM) and to the endomembrane system [7]. It is possible that NSvc4 is transported to the autophagosome from the ER after synthesis, or recycled from the PM.

Some viruses have evolved to protect their proteins from autophagic degradation. The γb protein of BSMV interacts with the autophagy-related protein ATG7 and block the processing of ATG8 to inhibit autophagic flux [28]. In our case, RSV infection in both rice and *N*. *benthamiana* upregulates the expression of genes from the type-I J-domain *NbMIP1* gene family (*N*. *benthamiana*) or *OsDjA5* (rice), and the subsequent accumulation of these MIPs promotes viral infection by protecting NSvc4 from autophagic degradation through direct interaction. Interestingly, the upregulation of *NbMIP1*s upon RSV infection is also caused by the UPR. The DTT or TM treatment can upregulate *NbMIP1*s (Fig 3F–3H), and the upregulation level of *NbMIP1*s is also decrease by 4-PBA treatment upon RSV infection (Fig 3I). We also found that TM treatment could promote the degradation of NSvc4 only upon silencing *NbMIP1*s (Figs 2H and 4J), which indicates that the UPR-induced autophagy does promote NSvc4 to be degraded, and the ER stress-related upregulation of NbMIP1s can protect NSvc4 from this autophagic degradation. This is another model for an arms race between RSV and host plants. RSV invasion elicits the UPR of plant cells. On the one hand, the UPR activates the autophagy pathway through which NSvc4 is degraded; on the other hand, the UPR will increase the production of NbMIP1s which is hijacked by NSvc4 to protect itself from autophagic degradation. Furthermore, the 4-PBA treatment inhibits the infection of RSV (S9 Fig), which is in agreement with previous results demonstrating that the UPR is required for the infection by plant viruses [12–14]. Since viruses are obligate parasites in their host cells, the UPR could reduce the negative impact of the viral infection, orchestrate the recovery of ER function, and in turn, benefit viral replication and infection. TBGp3, the MP of PVX, can also elicit the UPR in plant cells. Overexpressing TBGp3 led to localized necrosis, while co-expressing TGBp3 with BiP, a ER-resident chaperone, abrogated necrosis [15]. In this study, the accumulation of NSvc4 is regulated by autophagy and NbMIPs which both are elicited by the UPR. Therefore, we speculate that the UPR plays dual roles in the viral infection by fine-turning the accumulation level of NSvc4.

J-domain proteins often act as co-chaperones of HSP70, with both proteins functioning as a complex to help substrate folding [32]. Extensive studies have clearly shown the important roles of J-domain proteins in plant-virus interactions. The CP of potyviruses interacts with the tobacco J-domain protein NtCPIP and HSP70 to regulate the viral infection [37]. A J-domain protein from *N*. *benthamiana* interacts with PVX CP and the virus minus-strand RNA and regulates viral replication and movement [45]. TMV MP interacts with two different J-domain proteins, NbMPIP, and NbMIP1, which are required in the TMV infection cycle [38,46]. Moreover, some J-domain proteins have been reported to inhibit viral infection. Silencing *GmHSP40*.*1* in soybean enhanced susceptibility of the plant to soybean mosaic virus [47]. NbMIP1s were reported to interact with the MP of tomato mosaic virus (ToMV) and TMV. Silencing *NbMIP1*s impairs the stability of TMV and ToMV MPs and inhibits the viral infection [38]. In the present study, we demonstrate that NSvc4 is degraded through the autophagy pathway; however, it was previously shown that TMV MP is ubiquitinated and delivered to the 26S proteasome to be degraded [48]. This fact implies that the NbMIP1s-mediated maintenance of MP stability might be different between RSV and TMV. Furthermore, Du *et al*. proved that the C-terminal zinc finger-CTD domain of NbMIP1.1a interacts with ToMV MP, which led the authors to speculate that NbMIP1s could bind to ToMV and TMV MP and deliver it to the HSP70 chaperone network. However, we found that it is the N-terminal part of NbMIP1.4b, which contains the J and the G/F domains, interacts with NSvc4 (Fig 3D). The J domain is the key structure of J-domain-containing proteins, and that structure enables the interaction with HSP70. A HPD motif within the J domain is essential for this: mutation of the motif impairs the interaction and deprives the ability of J domain proteins to stimulate the ATPase activity of HSP70 [32,34–36,40]. In our study, we found that NbMIP1.4b(ΔHPD) still maintained the ability to protect NSvc4 from degradation and rescue the trafficking of the movement-deficient PVX(Δp25)-eGFP (Figs 4K, 4L, 5K and 5L). Coincidentally, some studies have described J domain-independent functions of DNAJ proteins in different processes [49]. The DNAJ protein P58^IPK^ downregulates host antiviral defense by inhibiting PKR, a protein kinase. The HPD motif mutant of P58^IPK^ still maintains the ability to inhibit PKR kinase activity [50]. DnaJA1 enhances influenza A virus RNA synthesis both *in vivo* and *in vitro*, and the J domain of DnaJA1 is not required for this function. Mutation of J domain-deleted DnaJA1 is sufficient to increase viral RdRp activity [51]. We also found that transient expressing NbMIP1.4b reduced the number of NSvc4-eGFP and RFP-NbATG8f1 co-localized punctate structures (Fig 4H and 4I), suggesting NbMIP1s might inhibit NSvc4 to be transported to autophagosome, However, the exact mechanism remains to be determined.

**Fig 7:**
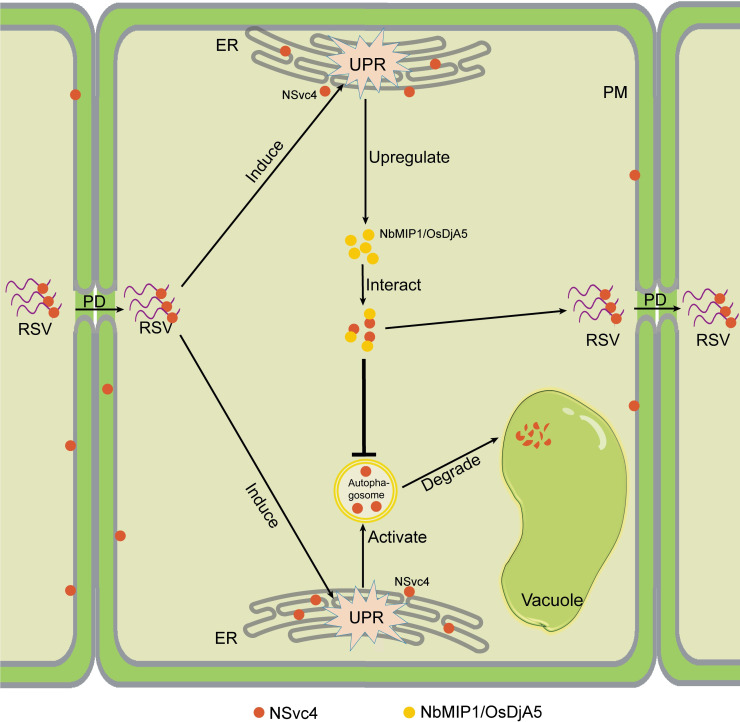
A model illustrates how NbMIP1s, autophagy, and the UPR regulate RSV infection. First, the ribonucleoprotein complex (RNP) of RSV enters a plant cell through PD. RSV infection elicits the UPR. Then, the autophagy pathway is activated by the UPR to promote the degradation of RSV NSvc4. As a counter-response, NSvc4 hijacks NbMIP1 family proteins (or OsDjA5 in rice) which are also upregulated by UPR, to protect NSvc4 itself from autophagic degradation. The stabilized NSvc4 binds the genome of RSV and facilitate it going through the PD to the next cell. However, the exact mechanisms about how NSvc4 is transported to autophagosomes and how NbMIP1s protect NSvc4 from autophagic degradation need further investigation. ER, endoplasmic reticulum; PM, plasma membrane; PD, plasmodesma.

The crosstalk between autophagy, ER stress, and the UPR has been previously demonstrated [52]. In our work, we explored the roles of UPR in RSV infection: RSV infection triggers the ER stress and the UPR. Autophagy is one of the downstream pathways of the UPR, aimed at cleaning up the excessive amount of aggregated proteins. In the process of RSV infection, activation of autophagy will target NSvc4 to degradation and restrict the virus cell-to-cell movement. As a counter-response, NSvc4 hijacks host J-domain proteins (NbMIP1s and OsDjA5), which are also upregulated by the UPR, to protect itself from degradation (Fig 7). Here, we present an example illustrating the arms race between RSV and the host plant, and how the host UPR plays dual roles in defense and viral infection through fine-tuning the accumulation of the virus-encoded NSvc4.

## Materials and methods

### Plant materials, agroinfiltration, and virus inoculation

All plants used in this study were grown in a growth chamber set at 25°C, 60% relative humidity, and a 16 h light and 8 h dark photoperiod. Five-leaf *N*. *benthamiana* plants were used for *Agrobacterium* infiltration, as described before [9]. RSV mechanical inoculation was described previously [8,9].

### Plasmid construction

cDNA of RSV-infected rice was generated by reverse transcription of total RNA, using ReverTra Ace qPCR RT Master Mix with gDNA Remover (TOYOBO, Osaka, Japan) according to the manufacturer’s instructions. Genes were cloned by PCR amplification with specific primers and fused into the corresponding vectors. For BiFC assays, NbMIP1s and NSvc4 were fused into plasmids carrying the N- or the C-terminal halves of YFP by T4 DNA ligase (Thermofisher, Waltham, the USA). The *in planta* expression vector pGD was digested by *BamH*I and *Sal*I, and NSvc4, NbMIP1.4b, and its mutants were fused into the vector using ClonExpress II One Step Cloning Kit (Vazyme, Nanjing, China) according to the manufacturer’s instructions. For hairpin construction, 300 to 600 bp forward and reverse sequences of the gene of interest, together with an intron from the *AtRTM1* gene of *Arabidopsis thaliana*, were fused into the pGD vector by in-fusion cloning. In order to induce the silencing of the entire gene family, 1–300 bp of *NbMIP1*.*4b* and 301–600 bp of *NbMIPL1* were fused together. For TRV-based VIGS, a 600 bp fragment combining 1–300 bp of *NbMIP1*.*4b* and 300–600 bp of *NbMIPL1* was constructed into the TRV RNA2 vector. The sequences of primers and genes are provided in S1 Table.

### Sequence and phylogenetic analyses

Sequences from yeast two-hybrid screening were sent to blastp searching in the *N*. *benthamiana* draft genome sequence database (https://solgenomics.net/organism/Nicotiana_benthamiana/genome/). Domain analysis was performed using InterProScan (http://www.ebi.ac.uk/InterProScan/).

Homologous genes of *NbMIP1s* were discovered by blastp searching in the rice genome in the NCBI database (http://www.ncbi.nlm.nih.gov/). Multiple full-length protein sequence alignment and Neighbor-Joining phylogenetic tree with 1000 bootstrap replicates were constructed using MEGA X software (https://www.megasoftware.net/).

### RNA isolation and real-time quantitative PCR

Total RNA was extracted from plant tissues using TRIzol reagent (Invitrogen, Carlsbad, USA). Primer pairs specific to target genes were designed by Oligo 7 (https://www.oligo.net/). RT-qPCR was performed using LightCycler 480 (Roche, Rotkreuz, Switzerland) as described previously [9].

### Western blotting and antibodies

Plant total protein extraction and western blotting were performed as described previously [53]. The membranes were probed with specific primary antibodies against FLAG tag (cat: F1804, Sigma, St. Louis, the USA), Myc tag (cat: A00704, GenScript, Piscataway, the USA), or Actin (cat: AC009, ABclonal, Wuhan, China). The rabbit polyclonal antibodies against RSV NSvc4 and NbMIP1.4b and the monoclonal antibody against RSV CP were generated in our lab. NbATG8-I and NbATG8-II were resolved by Tricine-SDS-PAGE with 16% resolving gel containing 6 M urea, followed by western blotting with a primary antibody against plant ATG8 (cat: AS14 2769, Agrisera, Vännäs, Sweden). Quantification of protein level in western blotting assay was performed using ImageJ software (https://imagej.nih.gov/ij/).

### Co-IP assay

Proteins were co-expressed in 4-week-old *N*. *benthamiana* leaves as shown in the figures. Total protein was extracted with the IP buffer containing 40 mM Tris-HCl, pH 7.5,150 mM NaCl, 5 mM MgCl2, 2 mM EDTA, 5 mM DTT, 0.1% Triton X-100, 5% glycerol, and EDTA-free protease inhibitor mixture (Roche, Basel, CH). Soluble proteins were cleared by centrifugation at 16,000 × g, 4°C for 15 min and then immunoprecipitated with Anti-FLAG M2 Magnetic Beads (Sigma–Aldrich, MO, USA). The Co-IP assay was then performed as described [9].

### Chemical treatments

Inhibitors of protein degradation pathways were infiltrated into plant leaves. MG132 (cat: HY-13259, MCE, Monmouth Junction, the USA), 3-MA (cat: HY-19312, MCE), and E64d (cat: HY-100229, MCE) (all dissolved in DMSO) were diluted in ddH_2_O to 100 μM, 5 mM, and 100 μM, respectively. An equal volume of DMSO was diluted in ddH_2_O as control. To inhibit protein synthesis, CHX (cat: HY-12320, MCE) dissolved in ethanol was added to 100 μM. After infiltration, leaves were moisturized for 6 h then harvested.

DTT was dissolved in ddH2O and diluted to 2 mM before infiltration into leaves. An equal volume of ddH2O was infiltrated as control. TM was dissolved in DMSO and diluted to 5 mg/mL before infiltration into leaves. An equal volume of DMSO diluted in ddH_2_O was infiltrated as control. 4-PBA was dissolved in ddH_2_O and diluted to 2 mM before infiltration into leaves. An equal volume of ddH_2_O was infiltrated as control.

### *In vivo* protein stability assay

NSvc4-FLAG was firstly expressed by agroinfiltration in *N*. *benthamiana* leaves. After 48 hours, 100 μM CHX was infiltrated into leaves, and half of the leaf was collected at this point as 0 h post CHX treatment. 4 h later, the other half of the leaf was sampled. Western blotting was then used to determine the NSvc4-FLAG protein level.

### *In vivo* protein degradation rate assay

Plant leaves expressing NSvc4-FLAG and hairpins to silence the genes of interest were infiltrated in ddH_2_O containing 100 μM CHX. Half of the infiltrated leaves was collected immediately after treatment, and then leaves were moisturized for 2 h before collecting the other half. Total proteins of the samples were extracted for western blotting detection.

### Disease severity index calculation

The symptoms of RSV-infected plants were monitored and documented in a time course. The severity of the disease was determined according to the following criteria: Level 1: only one leaf shows mild vein yellowing symptoms; Level 2: the area of vein yellowing expanded on the first leaf and symptoms begin to appear on the second leaf; Level 3: the vein yellowing symptoms almost occupy the whole area of the first and second leaves and appear on more leaves; Level 4: the vein yellowing area expanded on younger leaves, and leaves start curling; Level 5: significant symptoms show on all systemic leaves. Disease severity index (DSI) =  [sum (class frequency × score of rating class)]/[(total number of plants) × (maximal disease index)] .

### PVX movement complementation experiments

For PVX movement complementation experiments, a movement-deficient PVX-eGFP construct [PVX(Δp25)-eGFP] was kindly provided by Dr. Fei Yan (Ningbo University, China), and the assays were performed as previously described [9]. A fluorescence stereoscopic microscope was employed to observe the eGFP fluorescence at 5 dpi, and then the area of fluorescent signal was measured by ZEN lite software (Carl Zeiss, Germany). To minimize the influence of the differences to statistical analysis, at least 30 infection sites were measured.

### Subcellular localization assays and confocal microscopy

The eGFP fluorophore was excited at 488 nm and emission was detected at 490–540 nm. The YFP fluorophore was excited at 514 nm and emission was detected at 520–560 nm. The RFP fluorophore was excited at 561 nm and emission was detected at 560–620 nm. The chlorophyll was excited at 488 nm and emission was detected at 640–720 nm. The image were taken by laser scanning confocal microscope LSM 780 (Carl Zeiss) and processed with LSM software (Zen 2012, Carl Zeiss).

### Gene sequences and IDs

The nucleotide sequences or IDs of genes in this study were listed in S1 Text. Online genome database: Sol Genomics Network (https://solgenomics.net/) for *N*. *benthamiana*; Rice Genome Annotation Project (http://rice.plantbiology.msu.edu/) for rice.

## Supporting information

S1 Fig RSV infection activates the autophagy pathway of *N*. *benthamiana*. **(A)** Western blotting of NbATG8-I/-II in RSV-infected *N*. *benthamiana*. The total protein of RSV-infected plants and mock plants was extracted at 12 dpi. Western blotting was performed by using the antibody against NbATG8. Anti-RSV CP was used to indicate RSV infection. Actin was used as loading controls. **(B)** Confocal images of RFP-NbATG8f1 in RSV-infected and mock *N*. *benthamiana*. RFP-NbATG8f1 was expressed in the systemic leaves of RSV-infected and mock plants. Images were taken at 48 hpi by laser confocal microscopy. Bars, 50 μm. **(C)** Punctate spots of RFP fluorescence were calculated per cell. Numbers in RSV-infected and mock plants were analyzed by student’s *t*-test (two-sided, **P < 0.01). **(D)** Representative TEM images from local (6 dpi) and systemic (12 dpi) leaves of RSV-infected and mock *N*. *benthamiana*. Arrows indicate autophagic structures. Bars, 2 μm. Cp, chloroplast; CW, cell wall; N, nucleus; V, vacuole. **(E)** Numbers of the autophagic structures in RSV-infected and mock plants were analyzed by student’s *t*-test (two-sided, **P < 0.01).
(TIF)

S2 Fig Quantification of gene silencing efficiency. **(A)** RT-qPCR analysis of TRV-NbATGs inoculated *N*. *benthamiana*. The leaves from TRV-NbATG3, TRV-NbATG5, TRV-NbATG7, TRV-NbATG8, and TRV-NbTOR were harvested for total RNA extraction. One plant of each treatment was selected for RT-qPCR analysis to test the transcriptional level of *NbATG3*, *NbATG5*, *NbATG7*, *NbATG8c*, *NbATG8f*, and *NbTOR*. *NbActin* was used as an internal reference in relative quantification. The values represent the means ± SD of the expression levels relative to the TRV-GFP plant (*n* = 3 technical replicates). Student’s *t*-test was performed, and asterisks denote significant differences between TRV-NbATGs and TRV-GFP inoculated plants (two-sided, **P < 0.01, ***P < 0.001). **(B)** RT-qPCR analysis of NbATG5-hairpin or NbBeclin1-hairpin expressing leaves. NbATG5-hairpin, NbBeclin1-hairpin or GUS-hairpin were expressed in *N*. *benthamiana* leaves. The leaves were harvested at 60 hpi for total RNA extraction, and one leaf was selected for RT-qPCR analysis (*n* = 3 technical replicates) (two-sided, **P < 0.01). **(C)** RT-qPCR and western blotting analysis of NbATG8-hairpin expressing leaves. NbATG8-hairpin or GUS-hairpin were expressed in leaves of *N*. *benthamiana*. The experiment was performed as described above, and one leaf was selected for RT-qPCR analysis (*n* = 3 technical replicates) (two-sided, *P < 0.05, ***P < 0.001). Western blotting was performed to determine the protein accumulation of NbATG8 proteins. **(D)** RT-qPCR and western blotting analysis of NbMIP1-hairpin expressing leaves. NbMIP1-hairpin or GUS-hairpin was expressed in *N*. *benthamiana* leaves. The leaves were harvested at 60 hpi for total RNA and protein extraction. RT-qPCR was performed to determine the relative expression levels of *NbMIP1*s (*n* = 3 biological replicates) (two-sided, *P < 0.05, **P < 0.01, ***P < 0.001). Western blotting was performed to determine the protein accumulation of NbMIP1 family proteins.
(TIF)

S3 Fig Western blotting analysis of NbATG8 in autophagy-related genes silenced *N*. *benthamiana*. *NbATG3*, *NbATG5*, *NbATG7*, *NbATG8*, *NbTOR* were silenced by TRV-based VIGS. Total protein was extracted at 10 dpi and loaded to 16% Tricine gel with or without 6M urea. NbATG8 was detected by the antibody against plant ATG8.
(TIF)

S4 Fig Amino acid sequence alignment of NbMIP1 family proteins and domain prediction. **(A)** The amino acid sequence of NbMIP1s was aligned by the ClustalW method, and domains were discovered by InterPro scanning. **(B)** Phylogenetic tree of NbMIP1 family proteins. Sum of branch length = 0.543 is shown, and the percentage of replicate trees in which the associated taxa clustered together in the bootstrap test (1000 replicates) are shown next to the branches. The screened interacting protein of NSvc4 is named NbMIP1.2b and highlighted by red color text.
(TIF)

S5 Fig BiFC assay of NSvc4 with NbMIP1.4b(ΔHPD). The leaves of *N*. *benthamiana* were infiltrated with agrobacteria carrying NSvc4 and NbMIP1.4b(ΔHPD) fused to the N-terminal part or the C-terminal part of YFP, respectively. Samples were observed by laser confocal microscopy at 48 hpi. Bars, 50 μm.
(TIF)

S6 Fig Symptom of RSV-infected TRV-NbMIP1 and TRV-GFP pre-inoculated N. benthamiana. **(A)** 28 dpi; **(B)** 20 dpi. Bar, 1.0 cm.
(TIF)

S7 Fig The effect of NbMIP1.4b to PVX replication and protein expression. **(A)** PVX(Δp25)-eGFP and TBSV p19 was co-expressed with NbMIP1.4b-Myc or empty vector in each half of a leaf in *N*. *benthamiana*. **(B)** eGFP signal was observed by using a hand-held UV lamp at 48 hpi. **(C)** Western blotting of PVX CP and eGFP. The leaves in (B) were harvested at 48 hpi, and the total protein was extracted for western blotting. Actin was used as loading controls.
(TIF)

S8 Fig Homologues of NbMIP1s in rice interact with NSvc4 and are upregulated after RSV infection. **(A)** Phylogenetic tree of NbMIP1 and OsDjA family proteins. Sum of branch length = 5.37 is shown, and the percentage of replicate trees in which the associated taxa clustered together in the bootstrap test (1000 replicates) are shown next to the branches. **(B)** BiFC assay of NSvc4 with OsDjAs. The leaves of *N*. *benthamiana* were infiltrated with agrobacteria carrying NSvc4 fused to the N-terminal part of YFP and OsDjAs fused to the C-terminal part of YFP. Samples were observed by laser confocal microscopy at 48 hpi. Bars, 50 μm. **(C)** RT-qPCR analysis of *OsDjA* family genes expression level in RSV-infected or healthy rice. *OsUBQ* served as an internal reference in relative quantification. Values represent the means ± SD of the expression levels relative to the mock plants (*n* = 4 biological replicates). Data were analyzed using Student’s *t*-test, and asterisks denote significant differences between RSV-infected and mock plants (two-sided, *P < 0.05, **P < 0.01, n.s., not significant).
(TIF)

S9 Fig The effects of 4-PBA treatment to RSV infection. **(A)** The leaves were pre-treated with 4-PBA or ddH_2_O at 12 h before inoculating RSV. The severity of the symptom was observed at 20 dpi. Bar, 5.0 cm. **(B and C)** The diameters of chlorotic lesions on local leaves were measured (bar, 1.0 cm) at 6 dpi and analyzed by student’s *t*-test (two-sided, ***P < 0.001); the mean and the number of measured lesions (*n*) were labeled.
(TIF)

S1 Table DNA primers used in this study. (DOCX)

S1 Text The sequences and IDs of the genes in this study. (DOCX)
