# Supplementary figures and images for: The unfolded protein response plays dual roles in rice stripe virus infection through fine-tuning the movement protein accumulation
Source: PLoS Pathog. 2021 Mar 4;17(3):e1009370. doi: 10.1371/journal.ppat.1009370 (PMC8075255; doi:10.1371/journal.ppat.1009370)

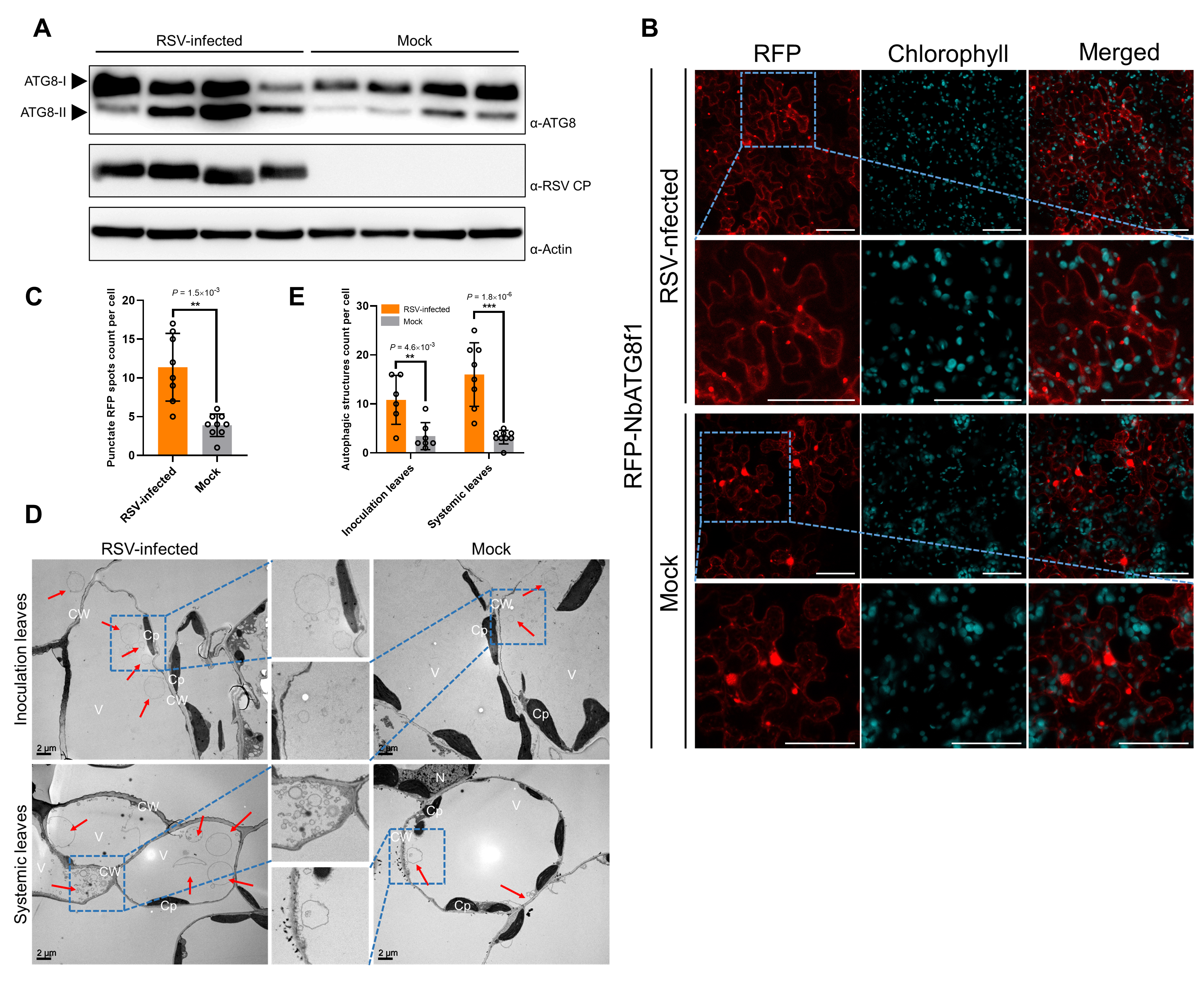

Supplement: S1 Fig — (A) Western blotting of NbATG8-I/-II in RSV-infected N. benthamiana. The total protein of RSV-infected plants and mock plants was extracted at 12 dpi. Western blotting was performed by using the antibody against NbATG8. Anti-RSV CP was used to indicate RSV infection. Actin was used as loading controls. (B) Confocal images of RFP-NbATG8f1 in RSV-infected and mock N. benthamiana. RFP-NbATG8f1 was expressed in the systemic leaves of RSV-infected and mock plants. Images were taken at 48 hpi by laser confocal microscopy. Bars, 50 μm. (C) Punctate spots of RFP fluorescence were calculated per cell. Numbers in RSV-infected and mock plants were analyzed by student’s t-test (two-sided, **P < 0.01). (D) Representative TEM images from local (6 dpi) and systemic (12 dpi) leaves of RSV-infected and mock N. benthamiana. Arrows indicate autophagic structures. Bars, 2 μm. Cp, chloroplast; CW, cell wall; N, nucleus; V, vacuole. (E) Numbers of the autophagic structures in RSV-infected and mock plants were analyzed by student’s t-test (two-sided, **P < 0.01). (TIF) [file ppat.1009370.s001.tif]

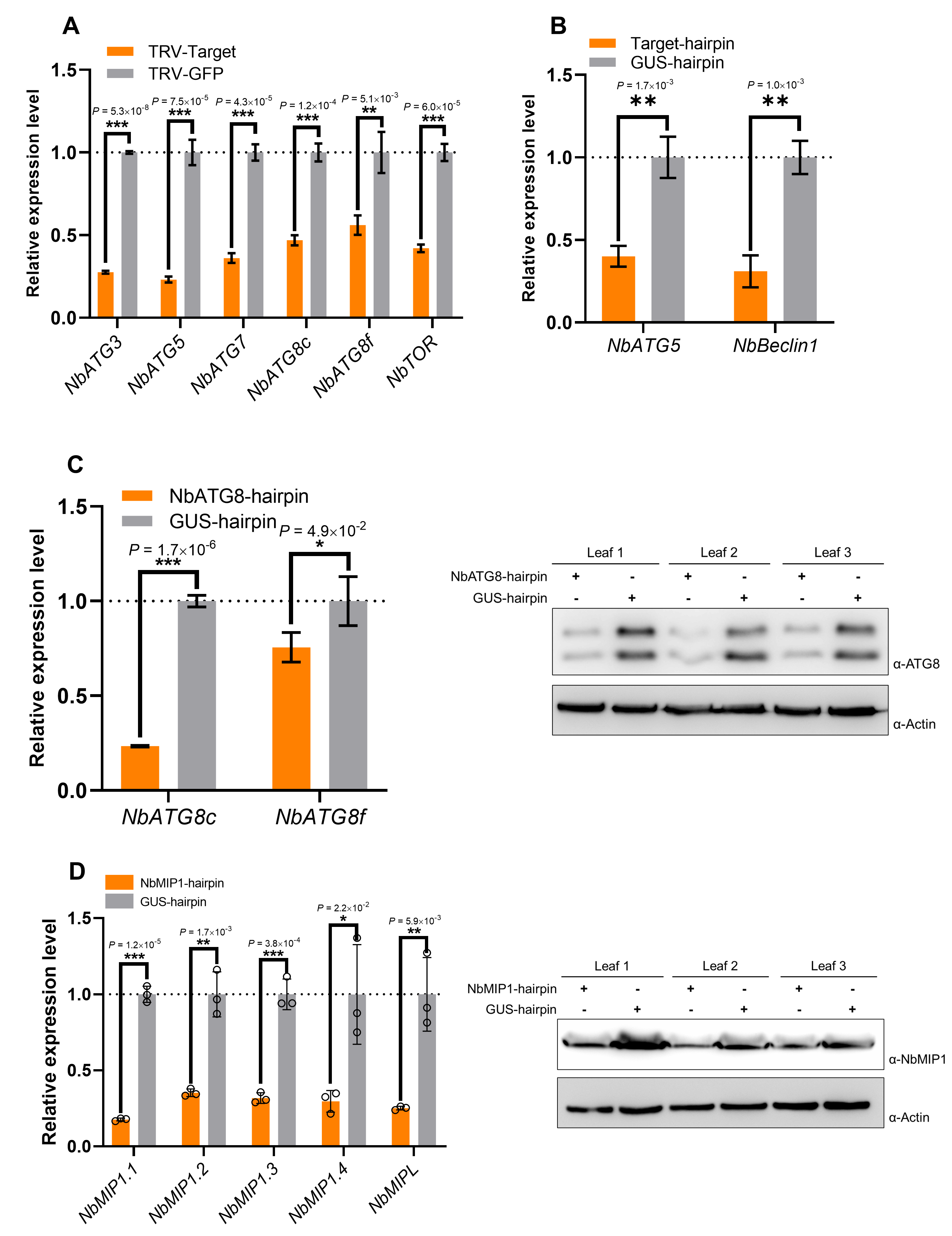

Supplement: S2 Fig — (A) RT-qPCR analysis of TRV-NbATGs inoculated N. benthamiana. The leaves from TRV-NbATG3, TRV-NbATG5, TRV-NbATG7, TRV-NbATG8, and TRV-NbTOR were harvested for total RNA extraction. One plant of each treatment was selected for RT-qPCR analysis to test the transcriptional level of NbATG3, NbATG5, NbATG7, NbATG8c, NbATG8f, and NbTOR. NbActin was used as an internal reference in relative quantification. The values represent the means ± SD of the expression levels relative to the TRV-GFP plant (n = 3 technical replicates). Student’s t-test was performed, and asterisks denote significant differences between TRV-NbATGs and TRV-GFP inoculated plants (two-sided, **P < 0.01, ***P < 0.001). (B) RT-qPCR analysis of NbATG5-hairpin or NbBeclin1-hairpin expressing leaves. NbATG5-hairpin, NbBeclin1-hairpin or GUS-hairpin were expressed in N. benthamiana leaves. The leaves were harvested at 60 hpi for total RNA extraction, and one leaf was selected for RT-qPCR analysis (n = 3 technical replicates) (two-sided, **P < 0.01). (C) RT-qPCR and western blotting analysis of NbATG8-hairpin expressing leaves. NbATG8-hairpin or GUS-hairpin were expressed in leaves of N. benthamiana. The experiment was performed as described above, and one leaf was selected for RT-qPCR analysis (n = 3 technical replicates) (two-sided, *P < 0.05, ***P < 0.001). Western blotting was performed to determine the protein accumulation of NbATG8 proteins. (D) RT-qPCR and western blotting analysis of NbMIP1-hairpin expressing leaves. NbMIP1-hairpin or GUS-hairpin was expressed in N. benthamiana leaves. The leaves were harvested at 60 hpi for total RNA and protein extraction. RT-qPCR was performed to determine the relative expression levels of NbMIP1s (n = 3 biological replicates) (two-sided, *P < 0.05, **P < 0.01, ***P < 0.001). Western blotting was performed to determine the protein accumulation of NbMIP1 family proteins. (TIF) [file ppat.1009370.s002.TIF]

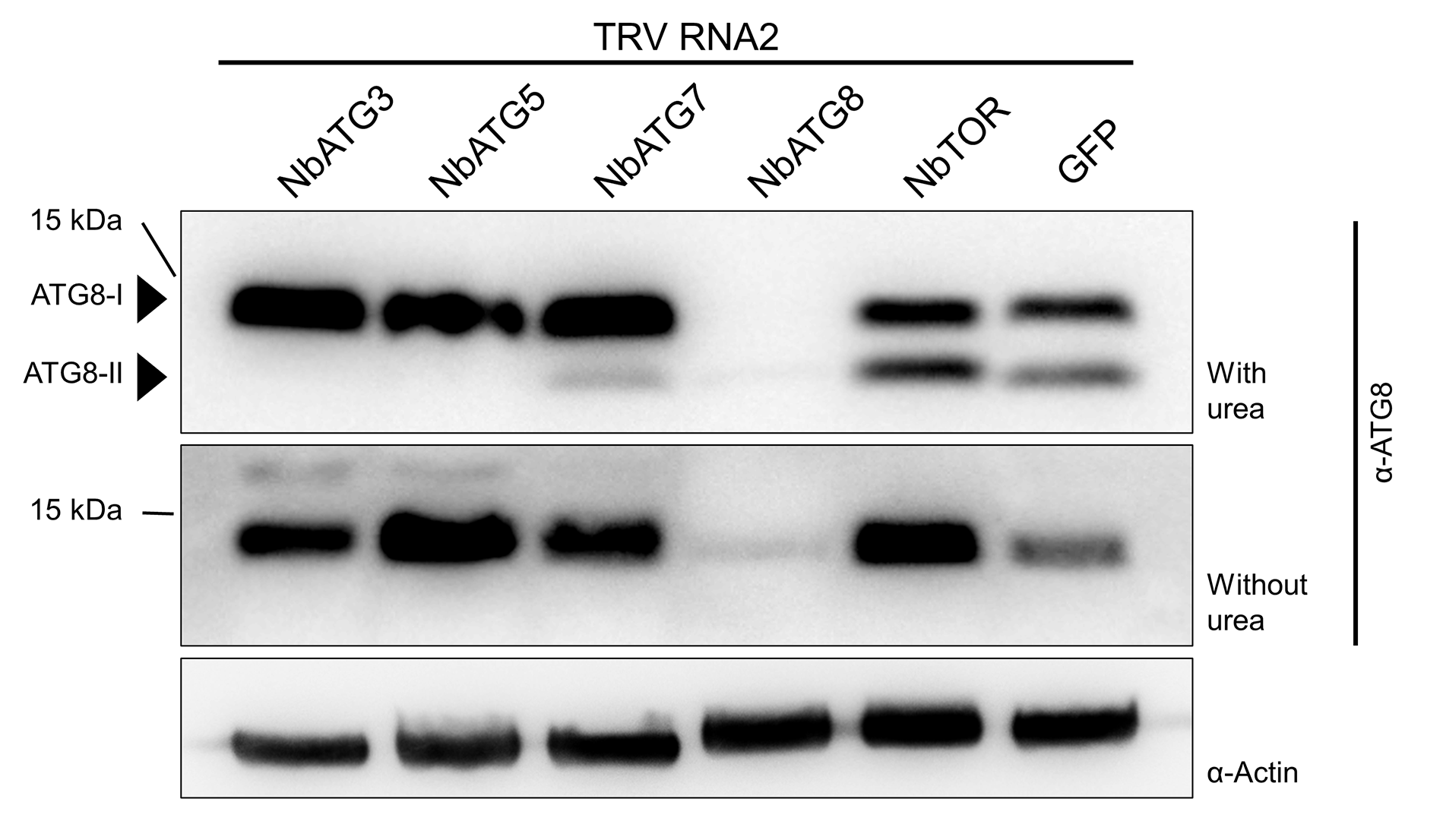

Supplement: S3 Fig — NbATG3, NbATG5, NbATG7, NbATG8, NbTOR were silenced by TRV-based VIGS. Total protein was extracted at 10 dpi and loaded to 16% Tricine gel with or without 6M urea. NbATG8 was detected by the antibody against plant ATG8. (TIF) [file ppat.1009370.s003.TIF]

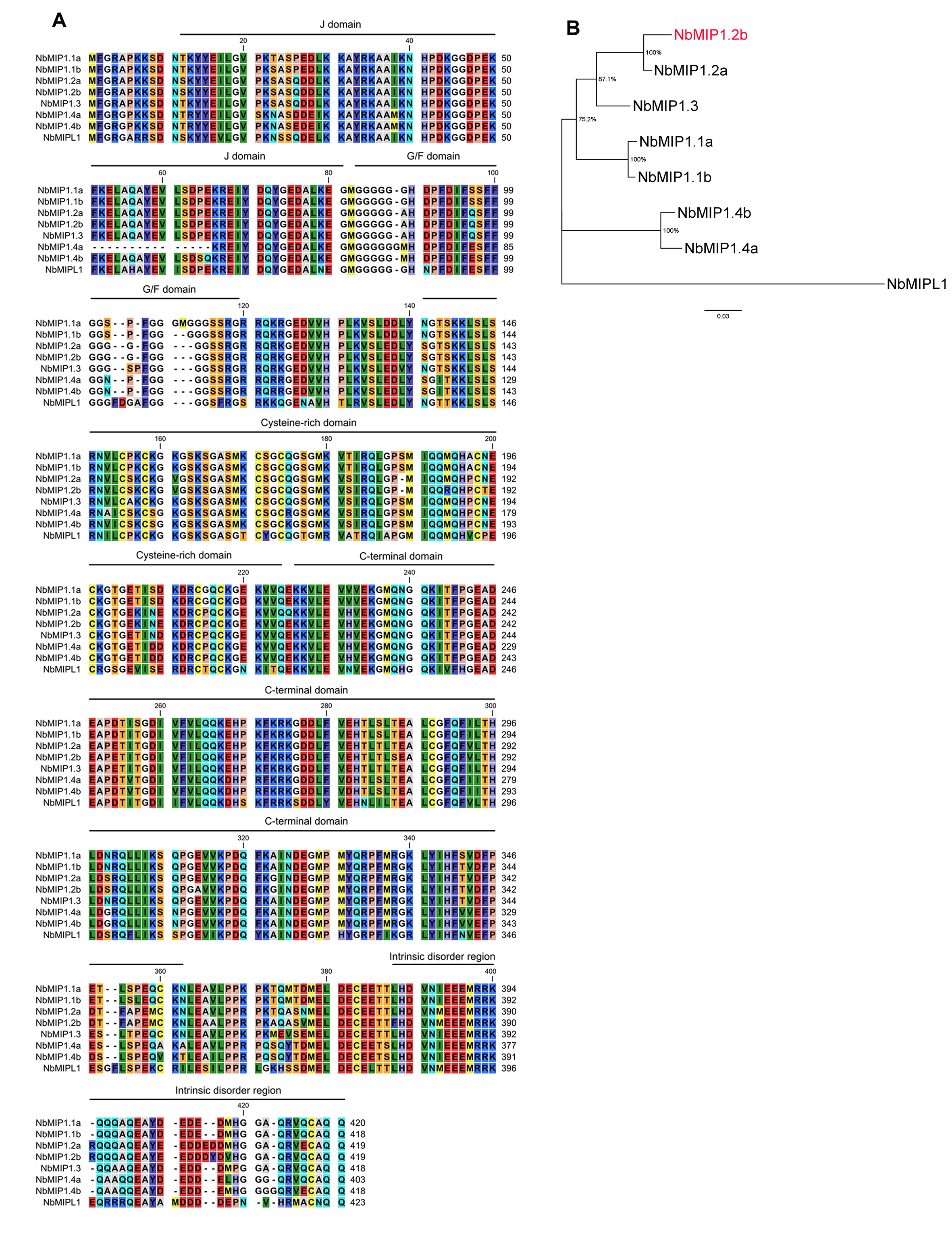

Supplement: S4 Fig — (A) The amino acid sequence of NbMIP1s was aligned by the ClustalW method, and domains were discovered by InterPro scanning. (B) Phylogenetic tree of NbMIP1 family proteins. Sum of branch length = 0.543 is shown, and the percentage of replicate trees in which the associated taxa clustered together in the bootstrap test (1000 replicates) are shown next to the branches. The screened interacting protein of NSvc4 is named NbMIP1.2b and highlighted by red color text. (TIF) [file ppat.1009370.s004.TIF]

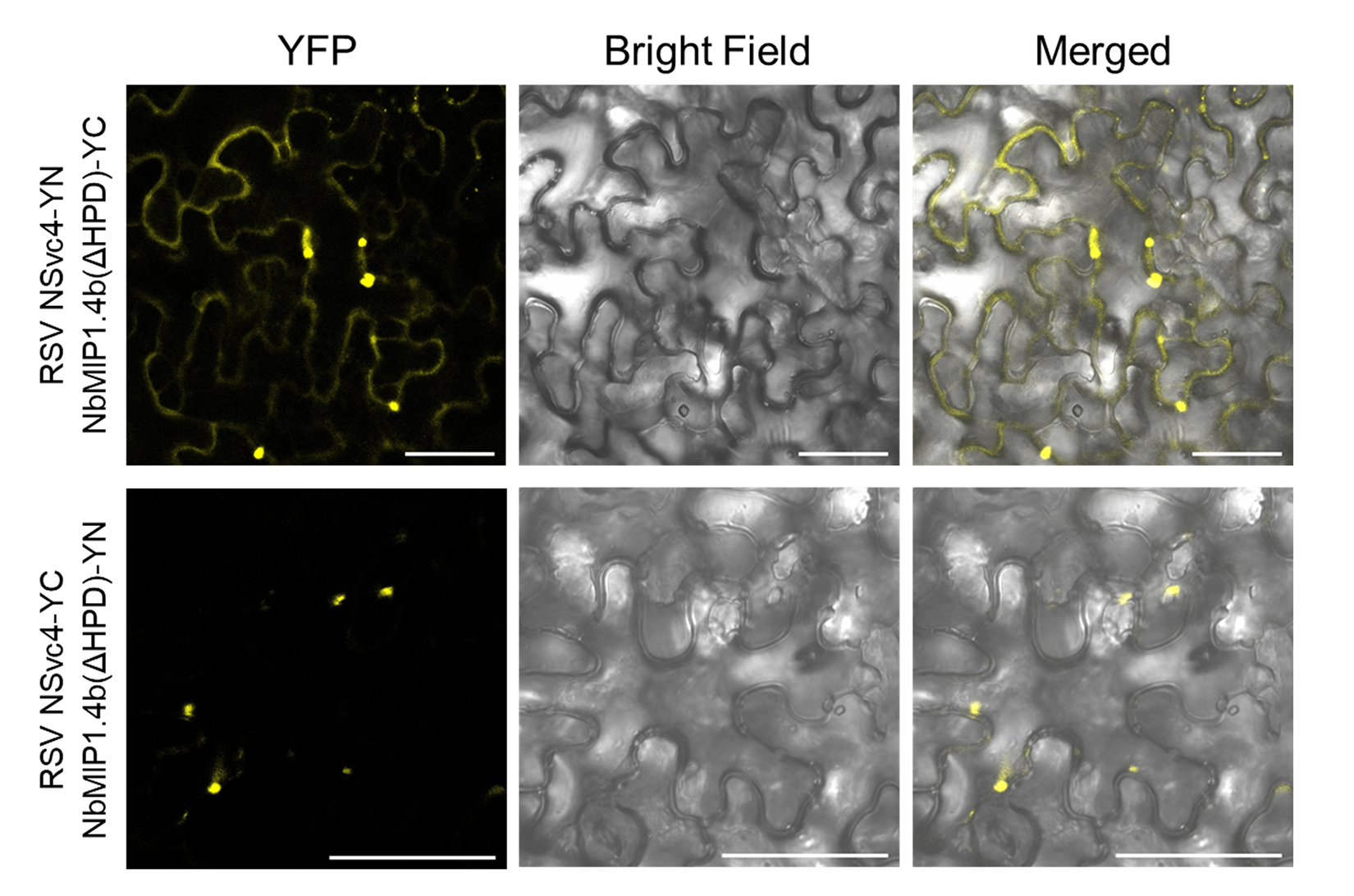

Supplement: S5 Fig — The leaves of N. benthamiana were infiltrated with agrobacteria carrying NSvc4 and NbMIP1.4b(ΔHPD) fused to the N-terminal part or the C-terminal part of YFP, respectively. Samples were observed by laser confocal microscopy at 48 hpi. Bars, 50 μm. (TIF) [file ppat.1009370.s005.TIF]

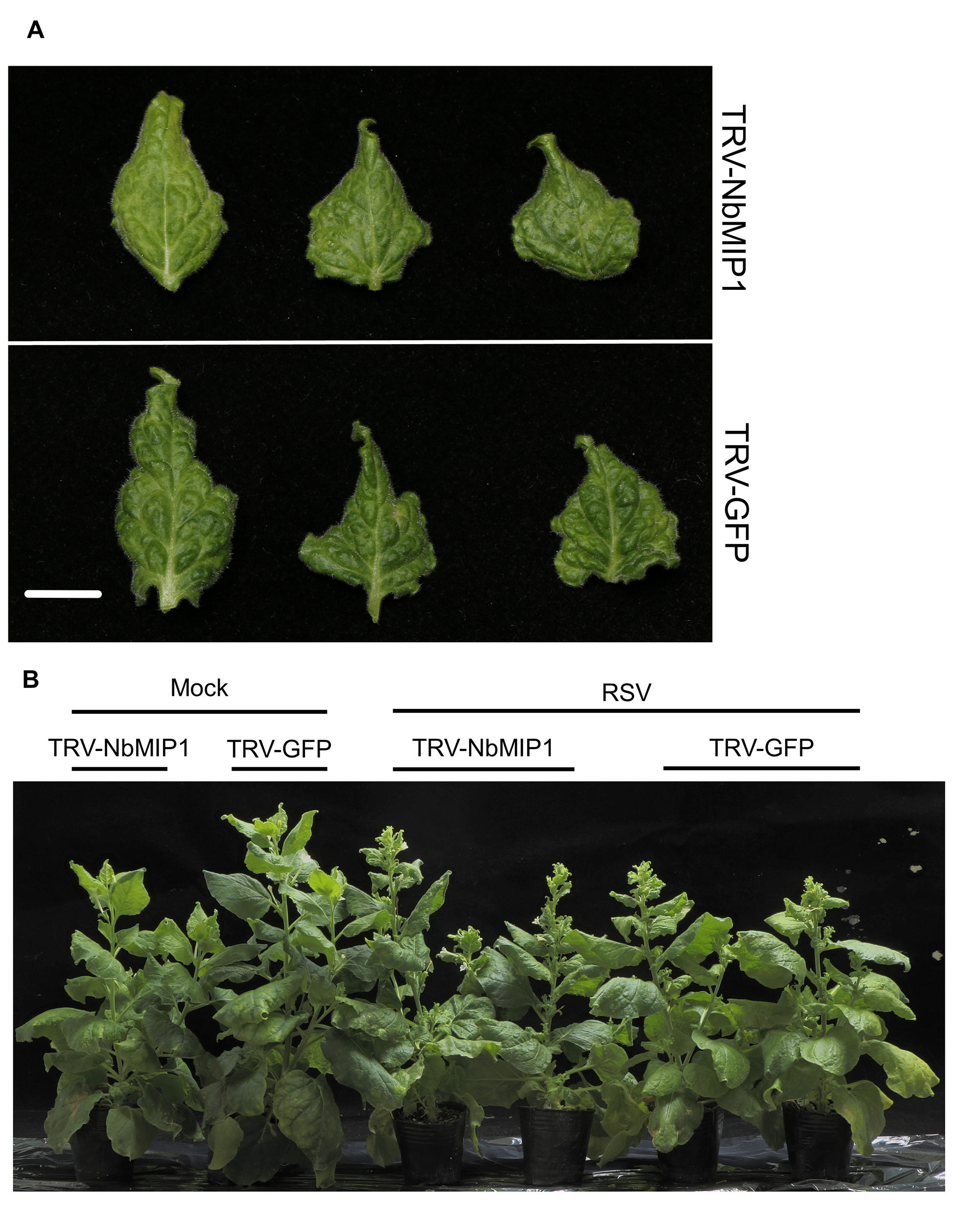

Supplement: S6 Fig — (A) 28 dpi; (B) 20 dpi. Bar, 1.0 cm. (TIF) [file ppat.1009370.s006.TIF]

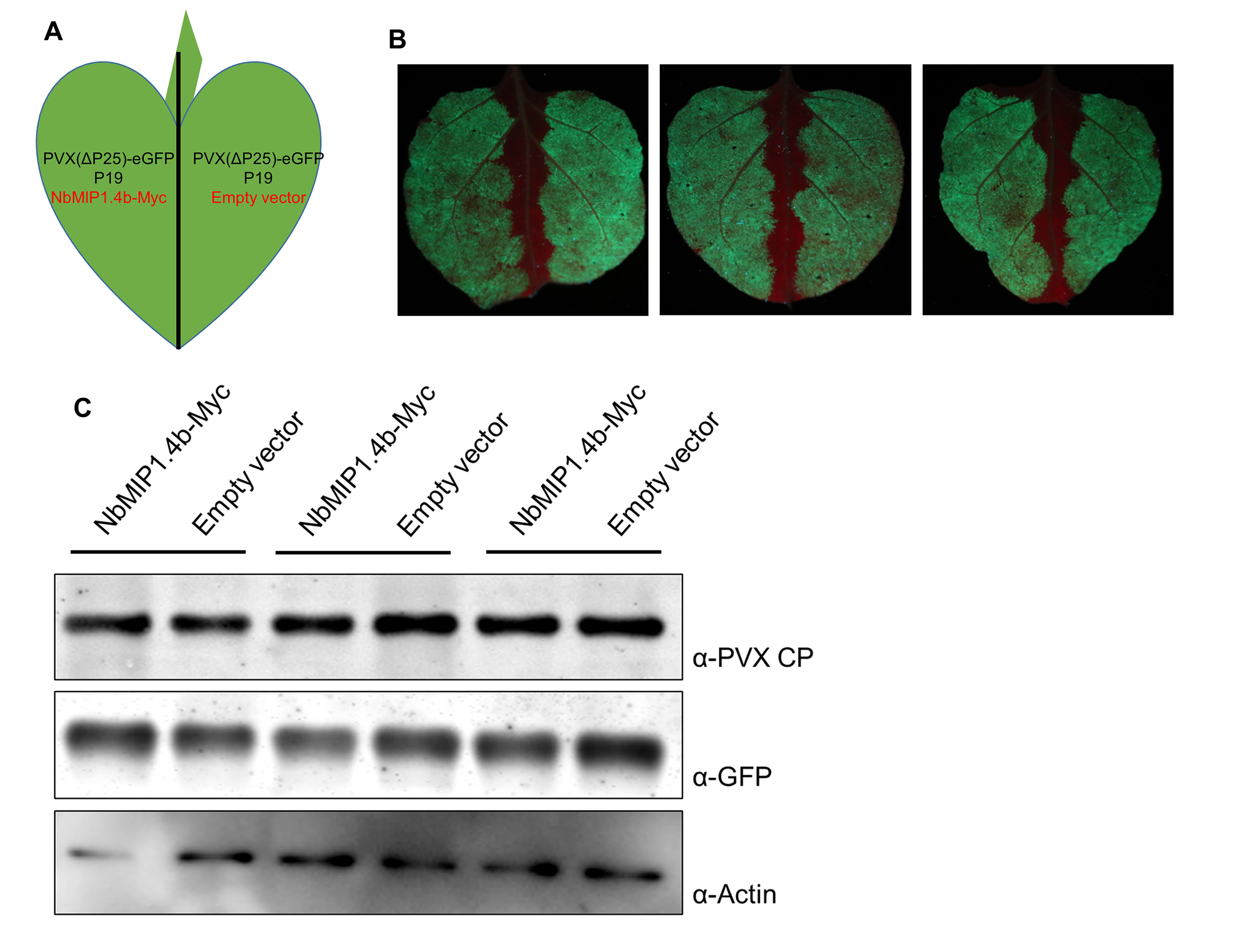

Supplement: S7 Fig — (A) PVX(Δp25)-eGFP and TBSV p19 was co-expressed with NbMIP1.4b-Myc or empty vector in each half of a leaf in N. benthamiana. (B) eGFP signal was observed by using a hand-held UV lamp at 48 hpi. (C) Western blotting of PVX CP and eGFP. The leaves in (B) were harvested at 48 hpi, and the total protein was extracted for western blotting. Actin was used as loading controls. (TIF) [file ppat.1009370.s007.TIF]

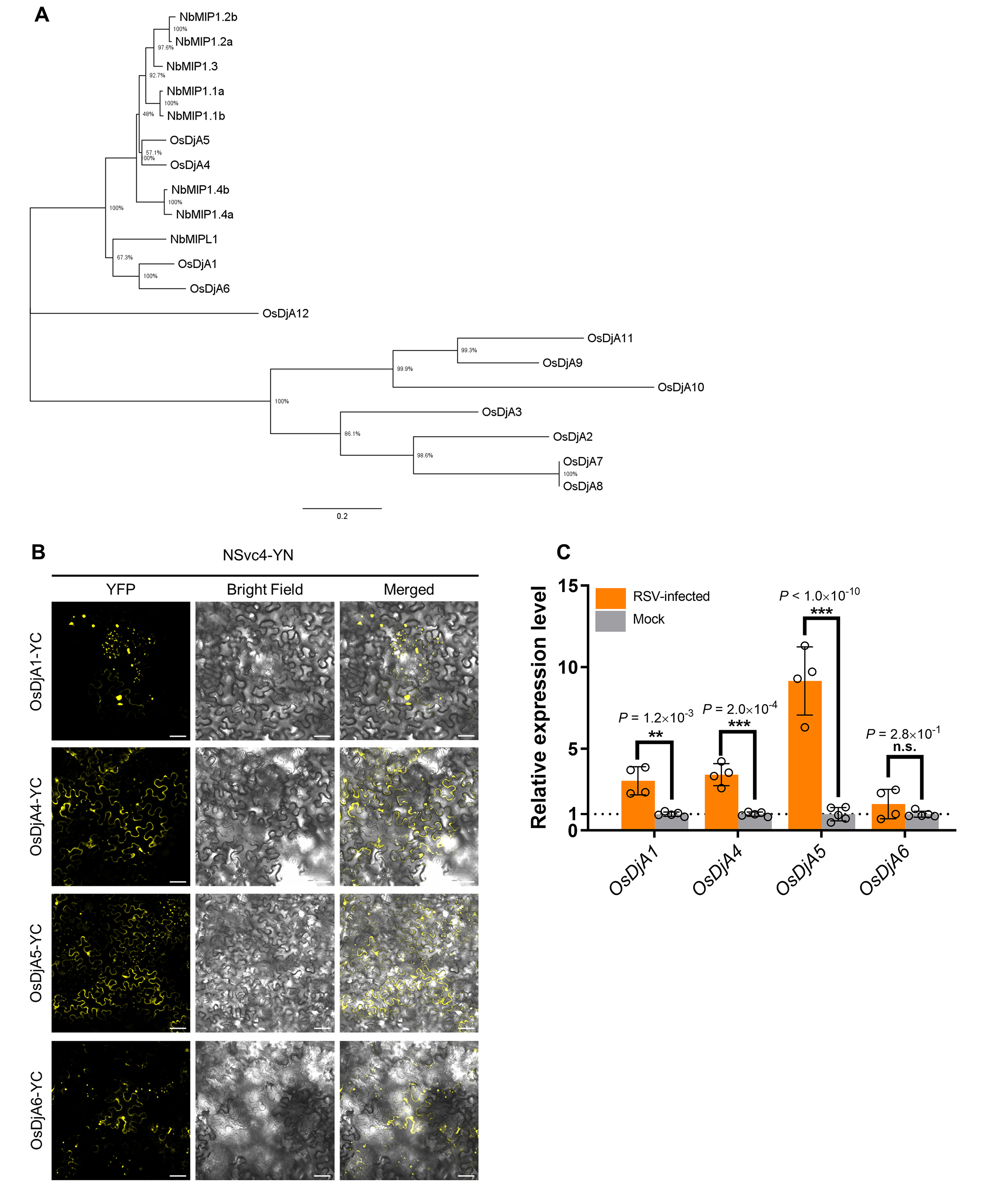

Supplement: S8 Fig — (A) Phylogenetic tree of NbMIP1 and OsDjA family proteins. Sum of branch length = 5.37 is shown, and the percentage of replicate trees in which the associated taxa clustered together in the bootstrap test (1000 replicates) are shown next to the branches. (B) BiFC assay of NSvc4 with OsDjAs. The leaves of N. benthamiana were infiltrated with agrobacteria carrying NSvc4 fused to the N-terminal part of YFP and OsDjAs fused to the C-terminal part of YFP. Samples were observed by laser confocal microscopy at 48 hpi. Bars, 50 μm. (C) RT-qPCR analysis of OsDjA family genes expression level in RSV-infected or healthy rice. OsUBQ served as an internal reference in relative quantification. Values represent the means ± SD of the expression levels relative to the mock plants (n = 4 biological replicates). Data were analyzed using Student’s t-test, and asterisks denote significant differences between RSV-infected and mock plants (two-sided, *P < 0.05, **P < 0.01, n.s., not significant). (TIF) [file ppat.1009370.s008.TIF]

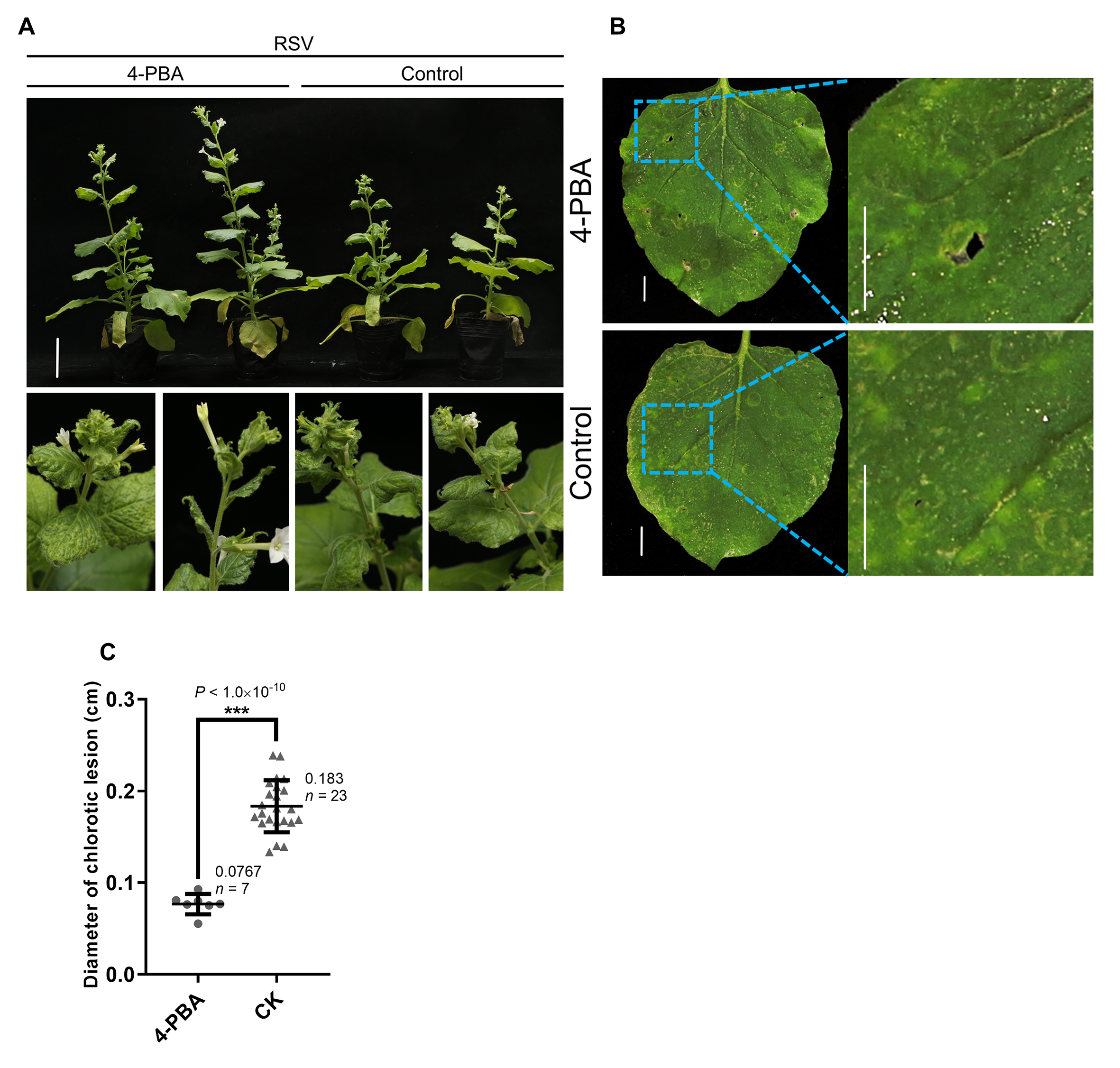

Supplement: S9 Fig — (A) The leaves were pre-treated with 4-PBA or ddH2O at 12 h before inoculating RSV. The severity of the symptom was observed at 20 dpi. Bar, 5.0 cm. (B and C) The diameters of chlorotic lesions on local leaves were measured (bar, 1.0 cm) at 6 dpi and analyzed by student’s t-test (two-sided, ***P < 0.001); the mean and the number of measured lesions (n) were labeled. (TIF) [file ppat.1009370.s009.TIF]
