# Supplementary material for: The unfolded protein response plays dual roles in rice stripe virus infection through fine-tuning the movement protein accumulation
Source: PLoS Pathog. 2021 Mar 4;17(3):e1009370. doi: 10.1371/journal.ppat.1009370 (PMC8075255; doi:10.1371/journal.ppat.1009370)
Supplement: S1 Table — (DOCX) [file ppat.1009370.s010.docx]

**S1 Table. DNA primers used in this study.**

| Gene cloning | Primer Sequence (5’-3’) | Purpose |
| --- | --- | --- |
| RSV NSvc4(BamHI)-F | ATGGATCCATGGCTTTGTCTCGACTTTT | cloning |
| RSV NSvc4(SalI)(NoStop)-R | ATGTCGACCATGATGACAGAAACTTCAG | cloning |
| NbMIP1.4b(XhoI)-F | CCGCTCGAGATGTTTGGGAGAGGACCAAAGAAG | cloning |
| NbMIP1.4b(KpnI)(NoStop)+1-R | GGGGTACCGCTGCTGGGCACATTCTACTCTCT | cloning |
| Os300(AACA)(BamHI)-F | ATGGATCCAACAATGTTCGGGCGCGCGCCGAA | cloning |
| Os300(SalI)(NoStop)+2-R | ACGCGTCGACATCTGCTGCGCGCACTGCACGC | cloning |
| NbMIP1.1a(PacI)-F | GGTTAATTAACATGTTTGGGAGGGCACCGAA | cloning |
| NbMIP1.1a(AscI)(NoStop)-R | TTGGCGCGCCCCTGCTGTGCACATTGAACTC | cloning |
| NbMIP1.2b(PacI)-F | GGTTAATTAACATGTTTGGCAGAGCACCAAA | cloning |
| NbMIP1.2b(AscI)(NoStop)-R | TTGGCGCGCCCCTGTTGTGCACATTGCACT | cloning |
| NbMIP1.4b(PacI)-F | GGTTAATTAACATGTTTGGGAGAGGACCAAAGAAG | cloning |
| NbMIP1.4b(AscI)(NoStop)-R | TTGGCGCGCCCCTGCTGGGCACATTCTACTCTCT | cloning |
| NbMIP1.4b_(1-134)(SgsI)-R | ATGGCGCGCCCACTGTAAAGATCCTCGAGAG | cloning |
| NbMIP1.4b_(135-418)(PacI)-F | CGTTAATTAACATGGGGATAACCAAGAAACT | cloning |
| NbMIP1.4b_(1-80)(SgsI)-R | ATGGCGCGCCCTTCTTTCAGTGCATCTTCTC | cloning |
| NbMIP1.4b_(81aa)(PacI)-F | CGTTAATTAACATGGGAATGGGTGGCGGCGGC | cloning |
| NbMIPL(PacI)-F | GGTTAATTAACATGTTTGGACGGGGTGCA | cloning |
| NbMIPL(AscI)(NoStop)-R | TTGGCGCGCCCTTGTTGGTTACAAGCCATACG | cloning |
| OsDjA1(PacI)-F | GGTTAATTAACATGTACGGACGCATGCCAA | cloning |
| OsDjA1(AscI)(NoStop)-R | TTGGCGCGCCCCTGCTGGGCACACTGTACC | cloning |
| OsDjA4(PacI)-F | GGTTAATTAACATGTTCGGGCGCGCGCCG | cloning |
| OsDjA4(AscI)(NoStop)-R | TTGGCGCGCCCCTGTTGCGCGCACTGAACTCT | cloning |
| OsDjA5(AscI)(NoStop)-R | TTGGCGCGCCCCTGCTGCGCGCACTGCACGCG | cloning |
| OsDjA6(PacI)-F | GGTTAATTAACATGTTTGGGCGTGTACCGAG | cloning |
| OsDjA6(AscI)(NoStop)-R | TTGGCGCGCCCCTGTTGAGCACACTGTACTCTT | cloning |
| AtRTM1 intron-F | ACGTTGTAAGTCTGATTTTTG | cloning |
| AtRTM1 intron-R | GCTCTATCTGCTGGGTCC | cloning |
| pGD+(BamHI)GUS600-F | tctacaaatctatctctGGATCCGTGGTGATGTGGAGTATTGCCA | cloning |
| GUS600+Intron-R | AAAAATCAGACTTACAACGTTCATTGTTTGCCTCCCTGCT | cloning |
| Intron+GUS600-F | TTGGACCCAGCAGATAGAGCTCATTGTTTGCCTCCCTGCT | cloning |
| GUS600(SalI)+pGD-R | atgtttgaacgagctctGTCGACGTGGTGATGTGGAGTATTGCCA | cloning |
| pLB+(BamHI)Fwr1.4b+L1-F | CTCGAGTTTTTCAGCAAGATGGATCCATGTTTGGGAGAGGACCAAAGAAG | cloning |
| Fwr1.4b+L1+Intron-R | AAAAATCAGACTTACAACGTTGAGCCTCGGCATTCAGGAC | cloning |
| Intron+Rev1.4b+L1-F | TTGGACCCAGCAGATAGAGCTGAGCCTCGGCATTCAGGAC | cloning |
| Rev1.4b+L1(SalI)+pLB-R | ATCTTCTAGAAAGATGTCGACATGTTTGGGAGAGGACCAAAGAAG | cloning |
| NbATG8f-1(BamHI)-F | ATGGATCCATGGCTAAGAGCTCATTCAAGC | cloning |
| NbATG8f-1(SalI)-R | ACGCGTCGACCTACAGCTTGTTCAGGTCCCC | cloning |
| NbMIP1(pTRV2)(XbaI)-F | gttaccgaattctctagaATGTTTGGGAGAGGACCAAAGAAG | cloning |
| NbMIP1(pTRV2)(KpnI)-R | acgcgtgagctcggtaccTGAGCCTCGGCATTCAGGAC | cloning |
| TRV-NbATG8C2I2(XbaI)-F | GCTCTAGAATGGCCAAGAGTTCTTTCAA | cloning |
| TRV-NbATG8C2I2(BamHI)-R | CGGGATCCTCAACTATTTGCACGACCAA | cloning |
| TRV-NbATG5(600bp)(XbaI)-F | GCTCTAGAATCTCGTCTAAGCTTAAACT | cloning |
| TRV-NbATG5(600bp)(KpnI)-R | GGGGTACCCTATATGGTGATGGGTTCTT | cloning |
| TRV-NbTOR(600bp)(XbaI)-F | GCTCTAGACTTCGTCTTCTTACTTTGTG | cloning |
| TRV-NbTOR(600bp)(KpnI)-R | GGGGTACCCTGTGTTAATTCAGCATCTTTA | cloning |
| NbATG3(pTRV2)(XbaI)-F | gttaccgaattctctagaAGAGCTGCATCAGTAGAGG | cloning |
| NbATG3(pTRV2)(KpnI)-R | acgcgtgagctcggtaccAGATGTGGATGGTCTTCAATG | cloning |
| NbATG7(pTRV2)(XbaI)-F | gttaccgaattctctagaTAGGCTTTGATAGCTTTCTAGTTA | cloning |
| NbATG7(pTRV2)(KpnI)-R | acgcgtgagctcggtaccAAGTACGAAGTCCATTCCTCT | cloning |
| NbActin-F | CAATCCAGACACTGTACTTTCTCTC | RT-qPCR |
| NbActin-R | AAGCTGCAGGTATCCATGAGACTA | RT-qPCR |
| NbMIP1.1_qRT-F | AAGCTGCTATTAAGAATCATCCTG | RT-qPCR |
| NbMIP1.1_qRT-R | TCCTTGAGAGCATCTTCACCGTA | RT-qPCR |
| NbMIP1.2_qRT-F | TTTGTAGAGCACACCTTGACC | RT-qPCR |
| NbMIP1.2_qRT-R | ATTCCTTCATCATTTATGCCCTT | RT-qPCR |
| NbMIP1.4_qRT-F | GCTCCAACAGAAAGACCAC | RT-qPCR |
| NbMIP1.4_qRT-R | TTGCCTTGAATTGATCAGGTT | RT-qPCR |
| NbMIPL1_qRT-F | TGGAATTTCCAGAATCTGGATTTC | RT-qPCR |
| NbMIPL1_qRT-R | ATAGCATAAGCCTCTTGCCTGC | RT-qPCR |
| NbbZIP60_qRT-F | ATTGACTCTAAGGACGGCTCT | RT-qPCR |
| NbbZIP60_qRT-R | ATACAACTTCTTCCGCTCTCG | RT-qPCR |
| NbBLP-4_qRT-F | TCGTTTTCGCAATCGTCCT | RT-qPCR |
| NbBLP-4_qRT-R | ATGTCCGTTCTTGTAGACACC | RT-qPCR |
| NbPDI_qRT-F | TTATTGCCAATCTTGACGCTGA | RT-qPCR |
| NbPDI_qRT-R | TTGCCCTTTCGAATCACGGCTA | RT-qPCR |
| NbCRT1_qRT-F | GGCTCCTTTGATTGACAACCC | RT-qPCR |
| NbCRT1_qRT-R | TGGCATACTCTGGATCATCGC | RT-qPCR |
| NbCAM13_qRT-F | AGCTGCAGGATATGATCACT | RT-qPCR |
| NbCAM13_qRT-R | CATTACATGCCTCAGCTCGTT | RT-qPCR |
| NbSKP1-1/-2_qRT-F | CTAGAGTCGCAGACAATCAAGCA | RT-qPCR |
| NbSKP1-1/-2_qRT-R | CGCTTGCAGTACTCAATCACC | RT-qPCR |
| NbSKP1-4_qRT-F | GTCCGCTTTGTTTCTCTCGTT | RT-qPCR |
| NbSKP1-4_qRT-R | CGTCGGAACTCTTCAACACA | RT-qPCR |
| NbbZIP17-1_qRT-F | TGGCTTGTTGTTCTTCATGCTC | RT-qPCR |
| NbbZIP17-1_qRT-R | AAAATTCTTCCGCGATGCCTT | RT-qPCR |
| NbbZIP17-2/-3_qRT-F | GCCTGCACCAAAGAGTAGCAA | RT-qPCR |
| NbbZIP17-2/-3_qRT-R | CCACTCATAAACGGTTCCCTC | RT-qPCR |
| NbATG3_qRT2-F | ATAATCTCGTCTCCAAATGCCCTA | RT-qPCR |
| NbATG3_qRT2-R | TGTCAGCAGGTAAATATGACTTCC | RT-qPCR |
| NbATG5_qRT2-F | GGCAGCATACATAATCAATGGGAA | RT-qPCR |
| NbATG5_qRT2-R | AATCATCCACTAATAGGCCAAG | RT-qPCR |
| NbATG7_qRT2-F | TCTTGTGATGGCTATTCCGAT | RT-qPCR |
| NbATG7_qRT2-R | CGACTTTCCCGTGTATCAGT | RT-qPCR |
| NbTOR_qRT-F | ACAGCAGCTCTTCAACCAG | RT-qPCR |
| NbTOR_qRT-R | AGTCTGAGATATACACGAGCAAC | RT-qPCR |
| NbATG8c_qRT-F | CACCCACTTGAAAGGCGACAGGC | RT-qPCR |
| NbATG8c_qRT-R | GCCTTCTCAGCACTAAGCTTTATTCTC | RT-qPCR |
| NbATG8d_qRT-F | AGTTAATACTTGTTCCTGCTGATCTGG | RT-qPCR |
| NbATG8d_qRT-R | ATTGCAGACGAATTCGCCAGAGTC | RT-qPCR |
| NbATG8h_qRT-F | TTCAGACGATGAGAGACTCGCAGAATC | RT-qPCR |
| NbATG8h_qRT-R | TTCCCAGGAGCCAGATGGAGTC | RT-qPCR |
| NbATG5_qRT2-F | GGCAGCATACATAATCAATGGGAA | RT-qPCR |
| NbATG5_qRT2-R | AATCATCCACTAATAGGCCAAG | RT-qPCR |
| NbBECN1_Qpcr-F | AACCGTTGTGTCTTGAATGC | RT-qPCR |
| NbBECN1_Qpcr-R | TGCTTCAAGTTTCCGCTCT | RT-qPCR |
| OsUBQ-F | ACCACTTCGACCGCACTACT | RT-qPCR |
| OsUBQ-R | ACGCCTAAGCCTGCTGGTT | RT-qPCR |
| OsDjA5_qRT-F | GCAACAGCAGCAGCAGGAG | RT-qPCR |
| OsDjA5_qRT-R | TTGTCGCCGCCGTCAAAG | RT-qPCR |
| OsDjA4_qRT-F | ATGAATGCGAGGAGACCACGAT | RT-qPCR |
| OsDjA4_qRT-R | GGCACCTCCAGGCATCTCA | RT-qPCR |
| OsDjA1_qRT-F | ACAGGCGGCAGGAAGCATA | RT-qPCR |
| OsDjA1_qRT-R | GTCCGAATGGCATCGAATTGTTCT | RT-qPCR |
| OsDjA6_qRT-F | GCAGTATCAAAGGAAGCAGGAAGC | RT-qPCR |
| OsDjA6_qRT-R | GGAGTATCATCACTTGCGCCAGA | RT-qPCR |
