# Supplementary material for: The unfolded protein response plays dual roles in rice stripe virus infection through fine-tuning the movement protein accumulation
Source: PLoS Pathog. 2021 Mar 4;17(3):e1009370. doi: 10.1371/journal.ppat.1009370 (PMC8075255; doi:10.1371/journal.ppat.1009370)
Supplement: S1 Text — (DOCX) [file ppat.1009370.s011.docx]

- **Cloned genes**

RSV NSvc4

ATGGCTTTGTCTCGACTTTTGTCCACTTCAAAAAGTAAGGTACTCTATGATGACCTTAGTGAGGAGTCCCAAAAGAGAGTTGATAATAAGAATAGGAAATCTCTAGCTCTCTCCAAGAGGCCTCTGAACCAGGGTAGGGTTACGATTGACCAAGCTGCCACAATGCTTGGATTGGAACCTTTCAGCTTCTCAGATGTCAAGGTTAACAAGTATGATATGTTCATAGCCAAACAGGACTATTCTGTGAAGGCCCATAGGAAAGCAACTTTCAACATACTTGTTGATCCATATTGGTTTCATCAACCTCTCACCCATTACCCATTCTTTAGAGTGGCAACTTTTGCTATGGTCTGGATTGGAATTAAGGGTAGAGCTAGTGGAATAACTACCCTCCGCATAATAGACAAGTCCTATGTGAACTCCTCAGATCAAGTTGAGGTAGAAGTTCGCTACCCAATTAGCAAGAATTTTGCTGTCTTGGGTTCTCTAGCGAATTTTCTGGCTTTGGAGGATAAGCATAATCTTCAAGTGTCCGTATCTGTTGATGACAGCTCAGTGCAGAACTGTGTCATCTCTAGAACTCTTTGGTTCTGGGGGATTGAGAGGACTGATCTGCCAGTGTCCATGAAGACTAATGACACTGTGATGTTTGAATTTGAACCACTAGAAGATAAAGCTATAAACCATTTGTCCAGCTTCAGTAATTTTACGACTAATGTTGTTCAGAAAGCTGTTGGAGGTGCTTTCACTAGCAAGAGCTTCCCTGAGCTAGACACTGAGAAGGAGTTCGGTGTGGTGAAGCAGCCAAAGAAGATACCCATCACAAAGAAATCAAAATCTGAAGTTTCTGTCATCATGTAG

NbMIP1.1a

ATGTTTGGGAGGGCACCGAAGAAGAGCGATAACACAAAGTACTATGAGATCTTAGGTGTTCCTAAGACGGCTTCACCTGAAGATCTCAAAAAAGCTTACCGTAAAGCTGCTATTAAGAATCATCCTGATAAGGGAGGTGATCCGGAAAAGTTTAAAGAGCTTGCACAAGCGTATGAGGTTTTGAGCGATCCGGAGAAGCGTGAGATATATGACCAGTACGGTGAAGATGCTCTCAAGGAGGGAATGGGTGGTGGGGGTGGTGGACATGACCCATTCGACATTTTCTCGTCTTTCTTTGGTGGCAGCCCGTTTGGCGGTGGTATGGGTGGTGGAAGCAGCAGAGGAAGAAGACAGAAAAGAGGAGAGGATGTTGTCCACCCTCTCAAAGTTTCTCTGGATGATCTGTACAATGGGACGTCAAAGAAGCTGTCACTATCCCGCAATGTATTGTGCCCCAAGTGCAAGGGGAAAGGGTCCAAGTCAGGTGCTTCAATGAAATGTTCTGGTTGTCAAGGGTCCGGGATGAAAGTCACTATTAGACAACTTGGTCCATCCATGATCCAGCAGATGCAGCATGCTTGTAACGAGTGTAAGGGCACTGGTGAGACAATCAGTGATAAAGATAGGTGTGGACAGTGTAAAGGTGAGAAGGTTGTGCAGGAGAAGAAGGTGTTGGAAGTTGTTGTTGAGAAGGGTATGCAGAACGGACAGAAGATTACGTTCCCGGGCGAGGCTGATGAAGCGCCTGATACTATCTCTGGAGACATAGTTTTTGTCTTGCAACAGAAGGAGCATCCCAAGTTCAAGCGAAAGGGTGATGATCTCTTTGTAGAGCACACTTTGAGTTTGACTGAGGCCCTATGTGGTTTCCAGTTCATCTTGACTCACTTAGACAATAGACAGCTACTCATTAAGTCCCAACCTGGAGAAGTTGTCAAACCTGATCAATTTAAGGCCATAAATGATGAAGGAATGCCGATGTACCAAAGGCCATTTATGAGAGGAAAACTGTACATTCACTTCTCTGTAGATTTCCCGGAGACATTATCCCCCGAGCAGTGCAAGAACCTTGAAGCGGTGTTGCCACCAAAACCCAAAACGCAAATGACTGATATGGAATTGGATGAGTGCGAGGAGACCACTTTGCATGATGTTAACATTGAAGAGGAGATGCGTAGGAAGCAGCAACAAGCCCAAGAGGCATATGACGAAGATGAAGACATGCATGGTGGCGCCCAAAGAGTTCAATGTGCACAGCAGTAA

NbMIP1.2b

ATGTTTGGCAGAGCACCAAAGAAGAGTGACAACTCGAAGTACTATGAGATATTAGGAGTTCCTAAGAGTGCTTCACAAGATGATCTGAAAAAAGCTTACCGTAAAGCCGCCATTAAAAATCATCCTGATAAGGGCGGGGATCCTGAAAAATTTAAGGAGCTTGCCCAAGCCTATGAGGTTTTGAGTGACCCAGAGAAGCGTGAGATTTATGATCAGTATGGTGAAGATGCACTTAAGGAAGGAATGGGTGGTGGAGGTGGGGCACATGACCCATTTGACATATTCCAGTCATTCTTTGGTGGCGGTGGATTTGGCGGTGGTGGAAGCAGCAGAGGAAGAAGGCAGAGGAAAGGGGAGGATGTTGTCCACCCTCTCAAGGTTTCTTTGGAGGATCTCTACAGTGGGACATCAAAGAAGCTATCTCTATCTCGCAATGTGTTGTGCTCAAAGTGCAAGGGAGTTGGGTCTAAATCAGGTGCTTCAATGAAATGTTCGGGCTGTCAAGGGTCTGGAATGAAAGTTTCTATCAGACAACTTGGCCCCATGATCCAGCAGAGGCAGCACCCTTGTACTGAGTGTAAGGGTACTGGTGAGAAAATCAACGAGAAAGATAGGTGCCCACAGTGTAAGGGTGAGAAAGTTGTGCAGGAGAAGAAGGTGTTGGAAGTTCATGTGGAGAAGGGTATGCAGAATGGACAGAAGATTACATTCCCAGGAGAGGCTGACGAAGCGCCCGAAACTATCACCGGGGACATAGTTTTTATATTACAACAGAAGGAACACCCCAAGTTTAAGCGCAAGGGAGATGATCTCTTTGTAGAGCACACCTTGACCTTGAGTGAGGCTCTATGTGGTTTCCAGTTCGTCTTGACTCACCTAGACAGCAGACAGCTGCTTATTAAATCCCAACCTGGAGCAGTTGTCAAGCCTGATCAGTTTAAGGGCATAAATGATGAAGGAATGCCAATGTACCAAAGGCCATTCATGAGGGGAAAACTGTACATTCACTTCACAGTGGACTTCCCCGATACATTTGCTCCAGAGATGTGCAAGAACCTTGAAGCAGCACTACCACCAAGGCCTAAAGCACAAGCGTCCGTTATGGAATTGGACGAGTGCGAGGAGACCACTTTTCATGATGTGAACATGGAAGAAGAGATGCGTAGGAAGAGGCAGCAGCAGGCCCAAGAGGCATACGAGGAAGACGATGACTACGATGTGCATGGTGGTGCGCAAAGAGTGCAATGTGCACAACAGTAA

NbMIP1.4b

ATGTTTGGGAGAGGACCAAAGAAGAGTGATAATACGAGGTACTATGAAATATTGGGTGTGCCAAAGAATGCATCAGAGGATGAAATCAAGAAAGCTTATAGAAAAGCTGCTATGAAGAATCACCCTGATAAGGGTGGTGACCCTGAAAAGTTTAAGGAGCTTGCTCAAGCTTATGAGGTTTTGAGTGACTCACAAAAGCGTGAGATTTATGATCAGTATGGAGAAGATGCACTGAAAGAAGGAATGGGTGGCGGCGGCGGAATGCATGATCCATTTGACATCTTTGAATCTTTCTTTGGTGGCAATCCGTTTGGAGGTGGTGGTAGCAGCAGAGGAAGAAGACAGAGAAGGGGTGAGGATGTAGTGCATCCACTGAAGGTCTCTCTCGAGGATCTTTACAGTGGGATAACCAAGAAACTGTCCCTTTCCCGCAATGTAATTTGCTCCAAGTGCAGTGGTAAAGGATCCAAGTCTGGTGCTTCAATGAAGTGTTCTGGTTGTAAAGGTAGTGGTATGAAGGTTTCAATTAGACAACTTGGCCCGTCAATGATCCAGCAAATGCAGCACCCTTGTAATGAATGCAAGGGTACTGGAGAGACTATTGACGATAAGGATCGGTGCCCTCAATGCAAAGGTGAAAAAGTGGTTCAGGAGAAGAAAGTCCTAGAAGTTCATGTTGAAAAAGGCATGCAAAATGGACAGAAAATTACATTCCCCGGAGAGGCTGATGAAGCGCCTGATACAGTTACTGGAGATATAGTTTTTGTGCTCCAACAGAAAGACCACCCAAGGTTCAAGAGAAAGGGTGATGATCTGTTTGTAGATCACACATTGAGTCTAACTGAGGCTTTATGTGGCTTCCAGTTCATAATAACACACTTGGATGGCAGACAACTCCTCATAAAATCTAATCCCGGGGAAGTTGTTAAACCTGATCAATTCAAGGCAATCAATGATGAGGGAATGCCAATGTATCAGAGGCCATTCATGCGGGGTAAATTGTACATTCATTTTGTCGTTGAATTCCCAGATTCATTGAGCCCAGAACAGGTTAAAACTCTCGAGGCAATTTTACCTCCAAGACCTCAATCACAGTACACAGACATGGAATTGGATGAGTGTGAGGAAACTTCATTACATGATGTGAATATTGAGGAGGAAATGAGAAGGAAACAAGCAGCCCAACAAGAGGCATATGATGAGGATGATGAGATGCATGGTGGTGGAGGACAGAGAGTAGAATGTGCCCAGCAGTAA

NbMIPL1 (Niben101Scf06436g04005.1)

ATGTTTGGACGGGGTGCAAGGAGGAGTGATAACTCCAAATACTATGAGGTTCTTGGTGTTCCAAAGAATTCCAGTCAAGATGAACTTAAAAAGGCGTATAGAAAAGCTGCTATAAAGAATCACCCTGACAAGGGTGGGGACCCTGAAAAATTCAAGGAATTGGCTCATGCATATGAAGTTATAAGTGATCCAGAGAAGAGAGAAATTTATGATCAGTATGGTGAAGATGCGCTTAATGAAGGAATGGGTGGGGGTGGTGGCGGTCACAACCCATTTGACATATTTGAGTCATTCTTTGGTGGAGGTTTTGATGGAGCTTTTGGCGGTGGTGGTAGCTTCAGAGGCAGCAGAAAGAAACAAGGTGAAAATGCGGTGCACACTCTACGGGTTTCTCTGGAAGACTTGTACAATGGCACAACCAAAAAGCTCTCTCTTTCACGGAATATACTGTGCCCAAAATGTAAAGGGAAAGGTTCAAAGAGTGGAGCTTCTGGAACATGTTATGGATGTCAAGGTACTGGAATGCGAGTCGCGACAAGACAGATAGCCCCAGGAATGATTCAACAGATGCAACATGTTTGTCCTGAATGCCGAGGCTCAGGAGAGGTTATAAGTGAGAGAGATAGGTGCACTCAGTGCAAGGGAAACAAAATTACACAAGAAAAGAAAGTATTGGAAGTGAATGTTGAGAAAGGGATGCAACACGGTCAGAAGATTGTTTTCCACGGGGAAGCTGATGAAGCTCCAGATACCATCACCGGCGATATTATTTTTGTATTACAACAGAAGGATCACTCGAAGTTCAGGCGAAAGTCTGATGATCTTTACGTGGAACACAATCTTATTTTGACAGAAGCTCTCTGTGGCTTTCAATTTGTTCTGACTCATCTTGACAGCAGGCAGTTTCTGATCAAATCTAGCCCCGGAGAAGTTATAAAGCCTGATCAATATAAGGCAATAAATGATGAAGGAATGCCCCATTATGGGAGGCCATTCATTAAGGGTCGGCTTTATATCCATTTTAATGTGGAATTTCCAGAATCTGGATTTCTTTCCCCCGAGAAATGCCGCATTCTTGAGAGTATTCTGCCACCGAGACTGGGGAAGCACTCGTCTGATATGGAGTTAGATGAGTGTGAGTTAACTACTTTGCATGATGTCAACATGGAGGAAGAAATGAGGCGCAAGGAGCAGCGACGCAGGCAAGAGGCTTATGCTATGGATGACGATGATGAGCCAAATGTGCATCGTATGGCTTGTAACCAACAATAA

NbATG8f1 (Niben101Scf13429g04022.1) (genebank: MG733106)

ATGGCTAAGAGCTCATTCAAGCAAGAGCATGATTTTGAGAAGAGGCGCGCTGAGGCTGCTCGGATTAGGGAAAAATACTCAGATAGGATTCCGGTGATAGTTGAAAAGGCTGAAAAAAGTGATATTCCCAACATCGACAAGAAAAAGTATCTCGTGCCAGCTGACTTGACAATTGGGCAATTTGTCTATGTCATTCGCAAGAGAATCAAATTGAGTGCAGAAAAGGCAATATTCATATTTATCGACAATGTCCTACCGCCAACAGGGGCAATCATGTCTGCAATCTATGACGAAAAGAAGGATGAAGATGGTTTCCTTTATGTTACTTACAGTGGAGAAAACACATTCGGGGACCTGAACAAGCTGTAG

OsDjA1 (LOC_Os02g43930.1)

ATGTACGGACGCATGCCAAAGAAGAGTAACAATACCAAGTATTATGAGGTGCTTGGTGTATCTAAGACAGCAACCCAGGATGAGCTGAAGAAAGCGTACCGTAAAGCTGCCATTAAAAACCACCCTGATAAGGGTGGAGACCCTGAGAAGTTTAAAGAATTGGCTCAAGCTTACGAGGTTCTTAATGATCCTGAAAAGAGGGAAATCTATGACCAATATGGCGAGGATGCACTCAAAGAAGGAATGGGAGGAGGCAGCAGCAGTGATTTCCATAGTCCCTTCGATTTATTTGAGCAAATTTTTCAGAATCGTGGTGGCTTTGGGGTAGGTAGAGGACACAGACAAAAGCGTGGCGAAGATGTGGTACATACTATGAAGGTTTCTTTAGAAGACCTGTATAATGGTACTACCAAAAAACTGTCTTTGTCACGGAATGCTCTGTGCACAAAGTGCAAGGGTAAAGGATCCAAGAGTGGGGCAGCAGCAACTTGCCATGGTTGTCATGGTGCAGGAATGAGAACAATAACAAGACAAATTGGGCTTGGCATGATCCAACAGATGAACACTGTTTGCCCTGAATGCAGAGGATCAGGTGAGATGATAAGTGACAAGGATAAATGCCCGAGTTGTAAGGGAAACAAAGTAGTCCAGCAGAAGAAGGTCTTGGAGGTTCATGTTGAGAAGGGAATGCAACATGGCCAAAAGATTGTATTCCAGGGTGAAGCTGATGAAGCTCCTGATACAGTGACAGGAGACATAGTTTTTGTCTTGCAACTTAAAGACCACCCAAAATTTAAGAGGAAGTTTGATGACCTCTTTACTGAGCACACAATCTCCCTGACCGAGGCTCTGTGTGGCTTCCAGTTTGTTCTAACCCATCTTGATGGTCGGCAACTCCTAATCAAATCTAATCCAGGGGAGGTTATAAAACCTGGTCAACACAAGGCCATCAATGATGAAGGCATGCCCCAGCATGGCCGCCCTTTCATGAAAGGTCGTCTTTTTGTTGAATTCAACGTGGAGTTTCCTGAGCCTGGTGCACTCACTCCTGGCCAATGCCGATCGCTTGAGAAGATTTTGCCACCACGACCCAGGAATCAATTGTCAGACATGGAGCTAGATCAATGTGAGGAGACCACCATGCATGATGTCAACATAGAAGAGGAGATGAGGCGCAGGCAGCAGCACAGGCGGCAGGAAGCATATGATGAAGACGACGACGAGGATGCTGGAGCTGGACCAAGGGTACAGTGTGCCCAGCAGTAA

OsDjA4 (LOC_Os03g44620.2)

ATGTTCGGGCGCGCGCCGAAGAAGAGCGACAACACCAAGTACTACGAGATCCTGGGGGTCCCCAAGACCGCCTCCCAGGACGACCTCAAGAAGGCGTACCGCAAGGCCGCCATCAAGAACCACCCCGACAAGGGCGGCGACCCCGAGAAGTTCAAGGAGCTTGCACAAGCTTATGAGGTATTGAGTGACCCGGAGAAACGTGAAATCTATGACCAATATGGTGAAGATGCCCTCAAGGAAGGAATGGGTGGAGGCGGATCCCATGTTGATCCATTTGACATCTTTTCATCATTCTTTGGACCTTCTTTTGGTGGTGGTGGCAGCAGCAGGGGCAGAAGGCAAAGGAGGGGAGAGGATGTGATCCATCCGCTTAAGGTTTCTCTAGAAGATCTTTACAATGGTACTTCAAAGAAGCTCTCTCTTTCCCGCAATGTCCTCTGCGCCAAGTGCAAGGGCAAGGGTTCCAAGTCTGGTGCTTCCATGAGGTGCCCAGGTTGCCAGGGGTCTGGCATGAAAATCACCATCCGCCAGCTGGGGCCTTCCATGATACAGCAGATGCAGCAGCCTTGCAATGAGTGTAAGGGGACTGGAGAGAGCATTAATGAGAAGGATCGCTGCCCAGGCTGCAAGGGCGAGAAGGTTATTCAGGAGAAGAAGGTTCTGGAGGTTCACGTTGAGAAGGGGATGCAACACAATCAGAAGATCACTTTCCCTGGTGAAGCTGATGAGGCGCCTGATACCGTTACGGGAGACATTGTATTCGTCCTCCAGCAGAAGGACCACTCCAAGTTCAAAAGGAAGGGCGATGATCTCTTTTATGAGCACACCTTATCTCTGACTGAAGCACTTTGTGGTTTCCAATTTGTCCTGACACATCTGGACAACAGACAGCTGCTCATTAAGTCAAACCCCGGTGAAGTTGTTAAGCCTGACCAATTCAAGGCAATAAACGATGAGGGAATGCCAATGTACCAGAGGCCTTTCATGAAGGGGAAGCTCTACATTCATTTCACGGTGGAGTTCCCTGATTCCCTGGCGCCTGAACAATGCAAGGCTCTCGAGGCTGTGCTTCCACCGAAGCCTGCATCCCAGCTGACAGAAATGGAGATAGATGAATGCGAGGAGACCACGATGCACGATGTCAACAACATTGAGGAAGAGATGCGCAGGAAAGCCCAAGCTGCTCAGGAGGCGTATGATGAGGACGATGAGATGCCTGGAGGTGCCCAGAGAGTTCAGTGCGCGCAACAGTAA

OsDjA5 (LOC_Os03g57340.1)

ATGTTCGGGCGCGCGCCGAAGAAGAGCGACAACACGCGGTACTACGAGGTGCTTGGGGTGCCCAAGGATGCGTCCCAGGATGACCTCAAGAAGGCGTACCGCAAGGCCGCCATCAAGAACCACCCCGACAAGGGCGGAGACCCCGAGAAGTTCAAGGAATTGGCTCAGGCTTATGAAGTCCTGAGTGACCCTGAGAAGCGTGAAATCTATGATCAGTACGGTGAAGATGCTCTCAAGGAGGGGATGGGTCCTGGTGGTGGGATGCATGACCCATTTGACATTTTTTCCTCATTCTTTGGAGGTGGCTTTGGAGGTGGTAGCAGTAGGGGCAGGAGACAGCGTAGGGGAGAGGATGTGGTTCACCCTCTGAAGGTTTCTCTGGAGGAATTGTACAATGGCACATCAAAGAAGCTCTCCCTTTCTCGCAATGTGCTCTGCTCCAAGTGCAATGGCAAGGGCTCGAAATCTGGTGCTTCCATGAAGTGCTCTGGTTGTCAAGGTTCTGGTATGAAGGTCCAAATTCGCCAGTTGGGGCCAGGAATGATTCAGCAAATGCAACATCCCTGCAATGAGTGCAAGGGAACTGGTGAGACCATCAGCGACAAGGATAGATGCCCAGGCTGCAAGGGTGAGAAGGTGGCGCAGGAGAAGAAGGTTCTTGAGGTGGTGGTCGAGAAGGGCATGCAGAATGGACAGAAGATCACCTTCCCTGGTGAGGCTGATGAAGCGCCCGATACTGTCACTGGAGACATTATCTTCGTCCTCCAGCAGAAGGAGCATCCCAAGTTCAAGAGAAAGGGAGATGACCTCTTCTACGAGCACACCCTGAACCTCACTGAGGCCCTTTGTGGCTTCCAGTTTGTTCTCACTCACTTGGACAACAGGCAGCTGCTTATCAAGTCCAAGCCCGGTGAAGTTGTCAAGCCTGATTCATTCAAGGCTGTCAACGACGAGGGCATGCCGATGTACCAGCGGCCATTCATGAAGGGGAAGCTCTACATCCACTTCTCCGTGGAATTCCCCGACTCTTTGAACCCTGACCAGTGCAAGGCCCTGGAGACCGTCCTCCCGCCAAGGCCGGTGTCGCAGTACACCGACATGGAGCTCGACGAGTGCGAGGAGACCATGCCGTACGACGTGAACATCGAGGAGGAGATGAGGAGGCGGCAGCAACAGCAGCAGCAGGAGGCATACGACGAGGACGAGGACATGCACGGCGGCGGCGCCCAGCGCGTGCAGTGCGCGCAGCAGTAA

OsDjA6 (LOC_Os04g46390.2)

ATGTTTGGGCGTGTACCGAGGAGTAACAACACCAAGTACTATGAGGTTCTTGGAGTTCCTAAAACTGCAAGCAAGGATGAGCTAAAGAAGGCATACCGGAAGGCTGCCATAAAAAACCATCCTGACAAGGGAGGGGATCCAGAAAAGTTTAAAGAATTATCACAAGCGTATGAGGTTCTCACTGATCCTGAGAAGAGAGACATATATGACCAATATGGGGAGGATGCTCTTAAGGATGGAATGGGAGGAGGCAGTGACTTCCATAATCCATTTGACATATTTGAGCAGTTTTTCGGGGGTGGTGCCTTTGGGGGGAGTAGCTCAAGAGTACGCAGACAGAGACGTGGTGAAGATGTGGCGCATACTTTGAAGGTGTCTTTAGAAGATGTGTATAATGGATCTATGAAGAAACTATCATTATCACGAAATATTCTGTGCCCAAAGTGCAAAGGAAAAGGGACCAAATCTGAGGCTCCAGCAACATGCTATGGTTGTCATGGTGTAGGAATGAGGAATATAATGCGACAGATAGGACTAGGCATGATTCAACATATGCAGACTGTCTGTCCTGAATGCAGAGGATCAGGTGAGATCATAAGTGACAGGGATAAATGCACAAACTGCAGAGCTAGCAAAGTTATTCAGGAGAAAAAGGTGCTTGAGGTTCATATTGAGAAGGGAATGCAACATGGCCAAAAAATTGTATTCCAAGGTGAAGCTGATGAAGCTCCTGATACAGTGACAGGAGATATAGTATTTATCTTGCAAGTTAAGGTACATCCAAGATTTAAGAGGAAATATGATGACCTGTTCATTGAGCGCACAATCTCTTTAACTGAGGCATTGTGTGGGTTCCAATTCATCCTCACTCATCTGGACAGTAGGCAGCTCCTAATCAAGGCAAATCCTGGCGAAATTATTAAACCTGGTCAACACAAGGCCATAAATGATGAGGGAATGCCACACCATGGCCGGCCTTTCATGAAGGGCCGTCTCTTTGTGGAATTCAATGTTGAGTTCCCTGAATCTGGTGTACTCTCCCGTGACCAATGCCGGGCACTTGAGATGATCCTACCACCTAAACCTGGGCACCAATTATCAGATATGGACCTGGATCAATGTGAGGAAACTACCATGCATGATGTGAACATAGAAGAGGAGATGAGGCGCAAGCAGTATCAAAGGAAGCAGGAAGCGTACGACGAAGATGAGGAGGAGGATGCTCCAAGAGTACAGTGTGCTCAACAGTAA

AtRTM1 intron

ACGTTGTAAGTCTGATTTTTGACTCTTCTTTTTTCTCCGTCACAATTTCTACTTCCAACTAAAATGCTAAGAACATGGTTATAACTTTTTTTTTATAACTTAATATGTGATTTGGACCCAGCAGATAGAGC

- **IDs of genes for RT-qPCR analysis**

NbATG5(Niben101Scf01320g03007.1)

NbBeclin1(Niben101Scf13308g00003.1)

NbATG8c(Niben101Scf08681g00003.1)

NbATG8f(Niben101Scf03374g01007.1)

NbATG3A1(Niben101Scf01204g00002.1)

NbATG3A2(Niben101Scf03644g020071)

NbATG3B1(Niben101Scf02420g00003.1)

NbATG3B2(Niben101Scf14587g00001.1)

NbATG5A(Niben101Scf01320g03007.1)

NbATG5B(Niben101Scf02433g01001.1)

NbATG7A(Niben101Scf06879g01005.1)

NbATG7B(Niben101Scf00165g03001.1)

NbTOR-1(Niben101Scf00953g08007.1)

NbTOR-2(Niben101Scf05710g03035.1)

NbbZIP60(Niben101Scf24096g00018.1)

NbBLP-4(Niben101Scf08590g00005.1) (Bip family protein)

NbPDI-1(Niben101Scf00332g04004.1)

NbPDI-2(Niben101Scf00466g04033.1)

NbCRT1(Niben101Scf00466g04036.1)

NbSKP1-1(Niben101Scf02658g00012.1)

NbSKP1-3(Niben101Scf06809g00029.1)

NbbZIP17-1(Niben101Scf32851g00038.1)

NbbZIP17-2(Niben101Scf03647g01004.1)

NbbZIP17-3(Niben101Scf00077g08013.1)
